# Supplementary material for: ZFT is the major iron and zinc transporter in Toxoplasma gondii
Source: eLife. 2026 Feb 5;14:RP108666. doi: 10.7554/eLife.108666 (PMC12875612; doi:10.7554/eLife.108666)
Supplement: Figure 1—source data 1. [file elife-108666-fig1-data1.zip › Figure 1 - Source Data 1. PDF file containing original western blots for Figure 1D, indicating the relevant bands and conditions/Figure 1_Source Data 1.pdf]

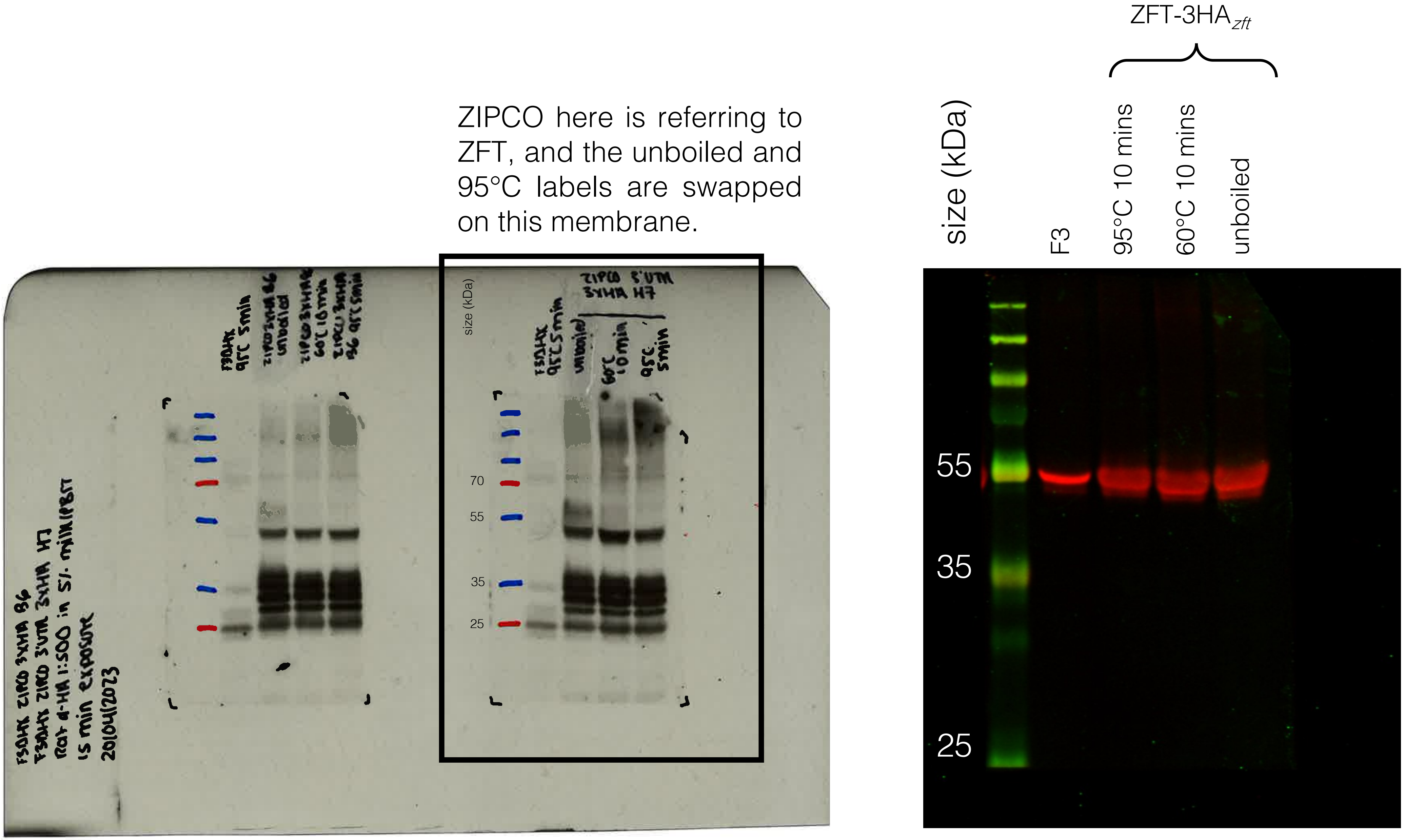

Figure 1, Source Data 1. Original membranes corresponding to Figure 1, panel D.
