# Supplementary figures and images for: ZFT is the major iron and zinc transporter in Toxoplasma gondii
Source: eLife. 2026 Feb 5;14:RP108666. doi: 10.7554/eLife.108666 (PMC12875612; doi:10.7554/eLife.108666)

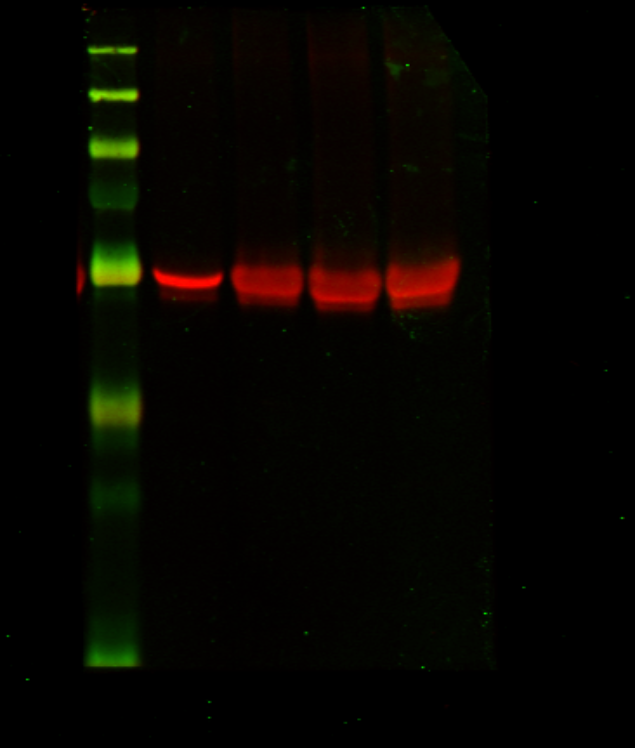

Supplement: Figure 1—source data 2. [file elife-108666-fig1-data2.zip › Figure 1 - Source Data 2. Original files for western blot analysis displayed in Figure 1D/F3dHX ZFT 3HA 3'UTR H7 CDPK1 Loading Control Staining.tif]

2A

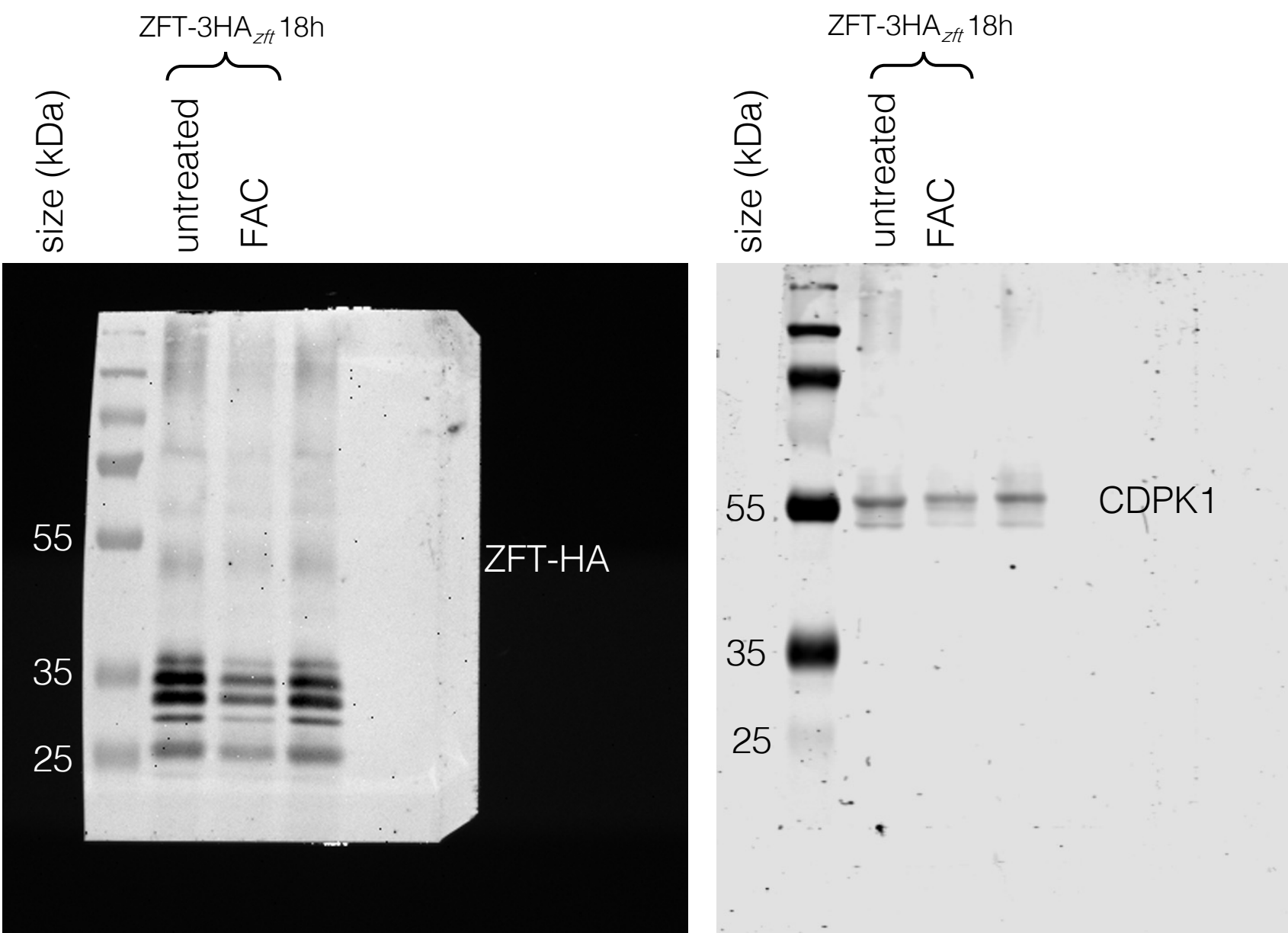

2C

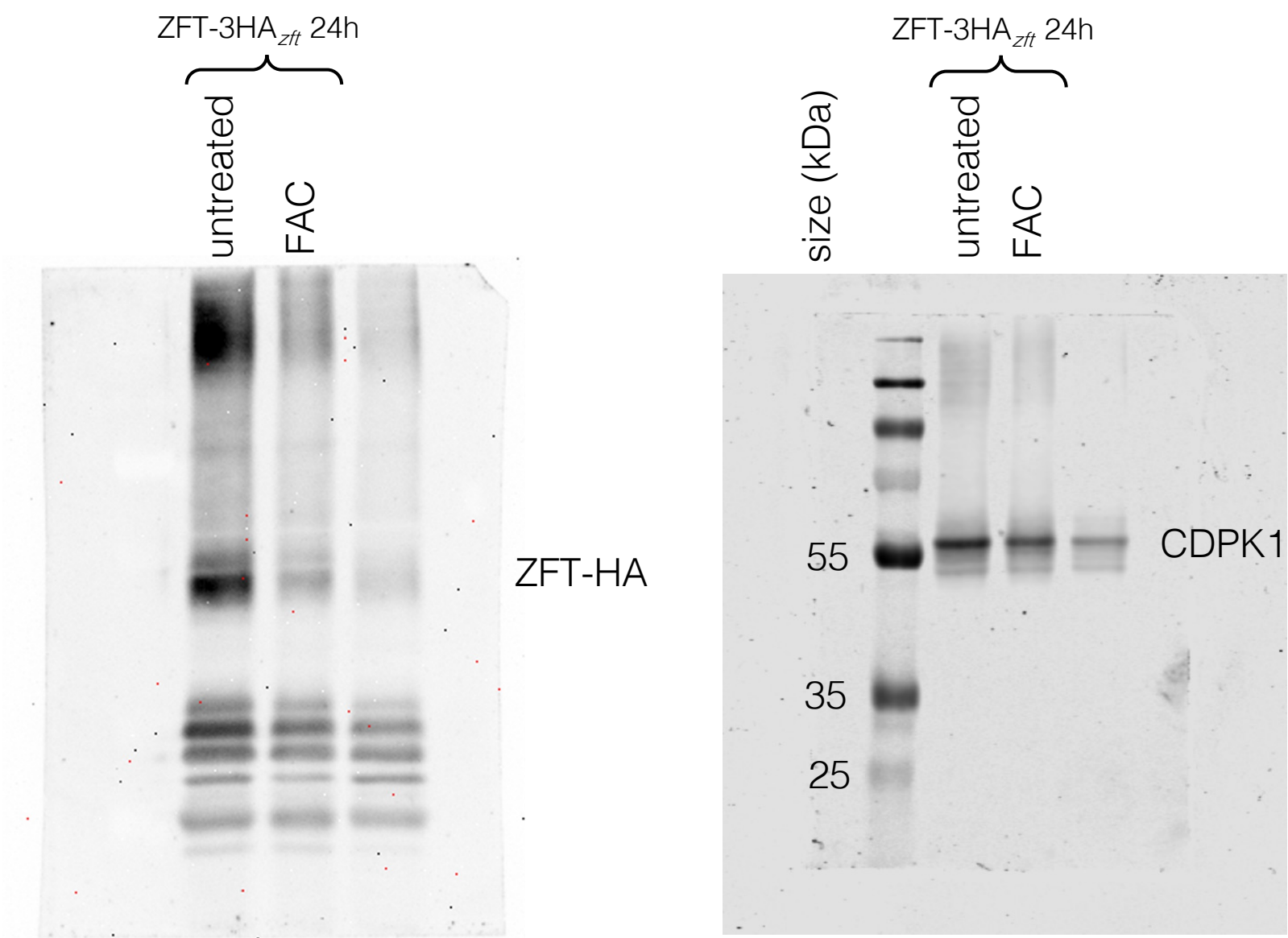

2H

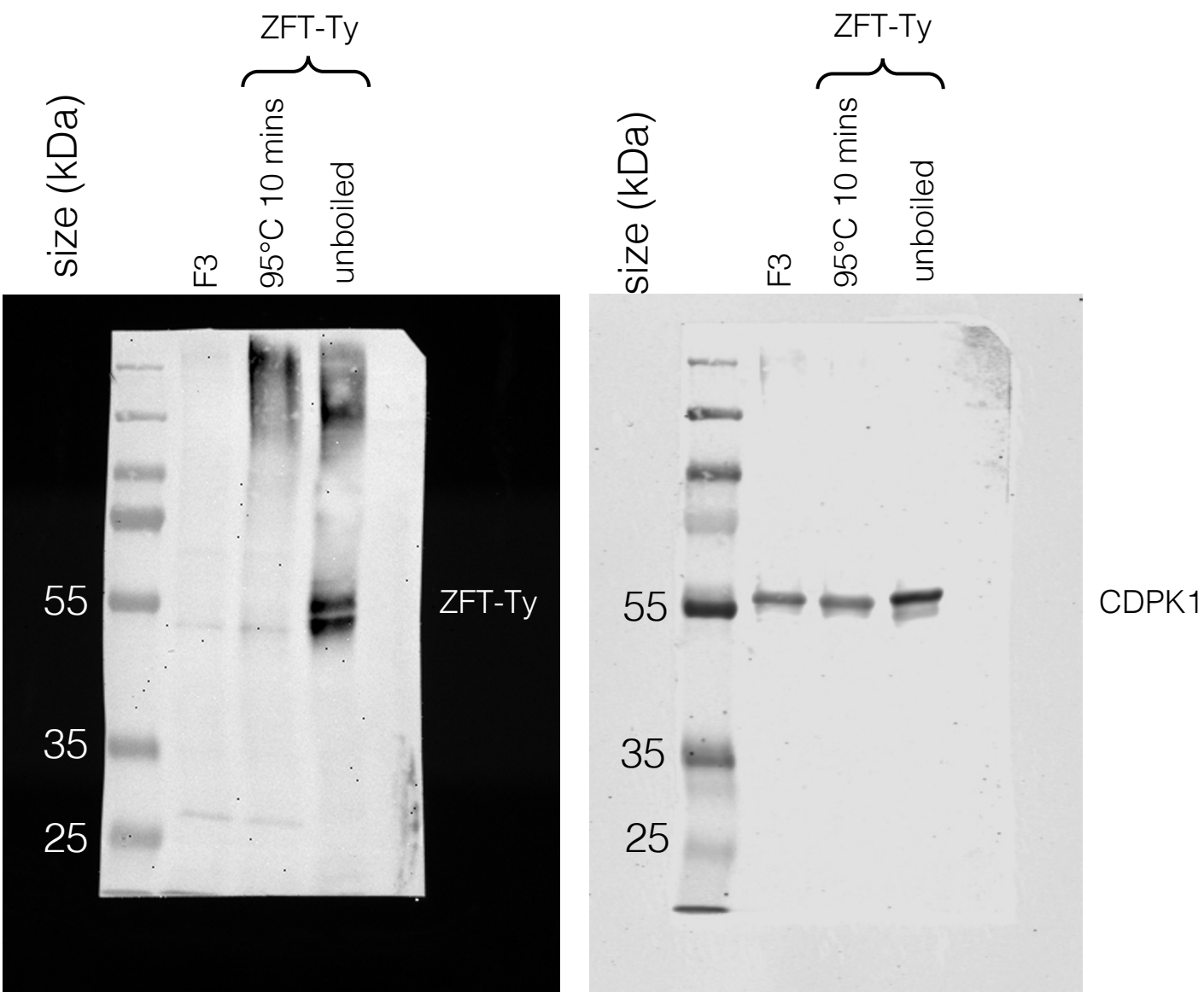

Figure 2, Source Data 1. Original membranes corresponding to Figure 2, panels A, C and H.

Supplement: Figure 2—source data 1. [file elife-108666-fig2-data1.zip › Figure 2 - Source Data 1. PDF file containing original western blots for Figures 2A,2C and 2H indicating the relevant bands and conditions/Figure 2_Source Data 1.pdf]

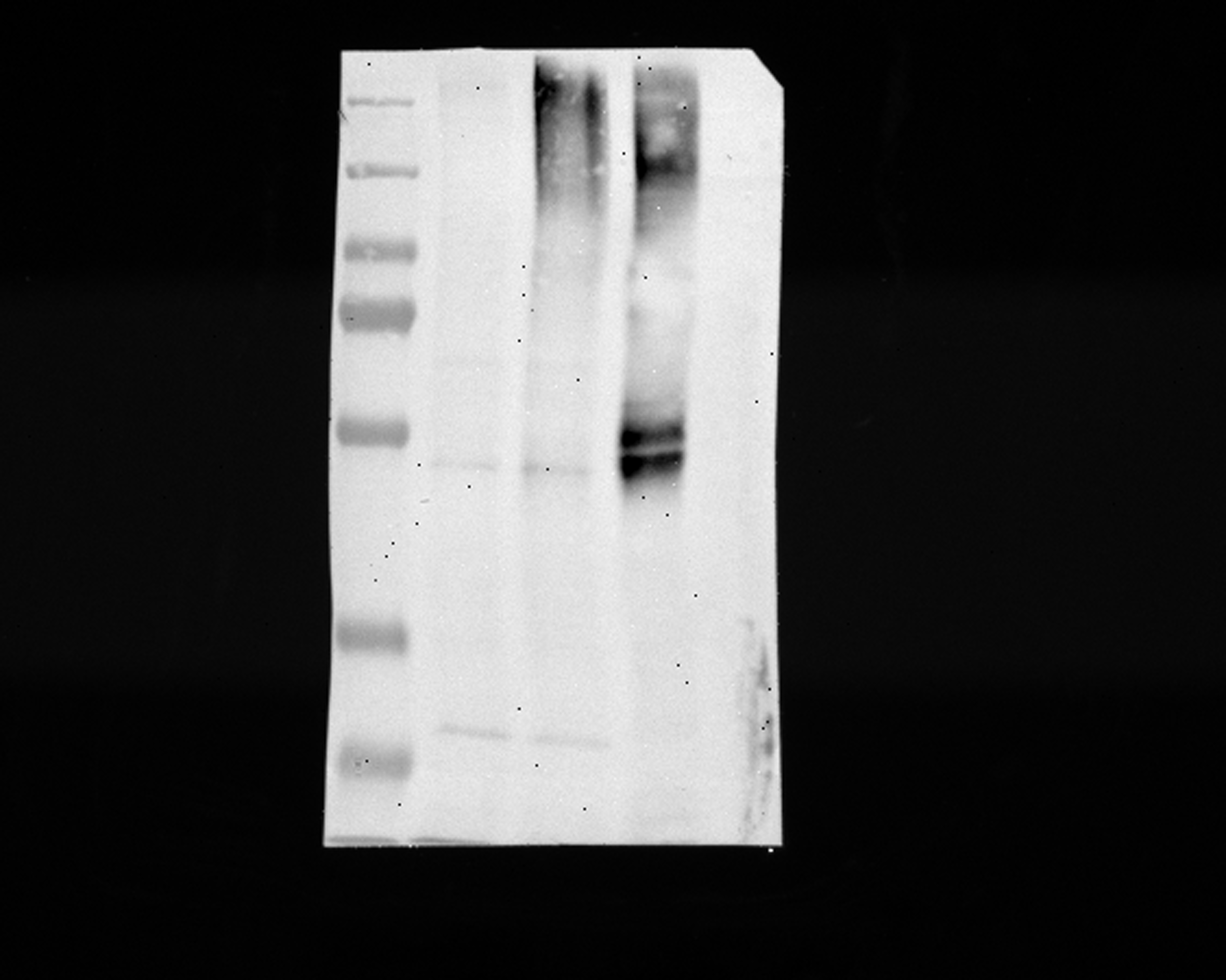

Supplement: Figure 2—source data 2. [file elife-108666-fig2-data2.zip › Figure 2 - Source Data 2. Original files for western blot analysis displayed in Figures 2A, 2C and 2H/CHEMI_11302023_134440.tif]

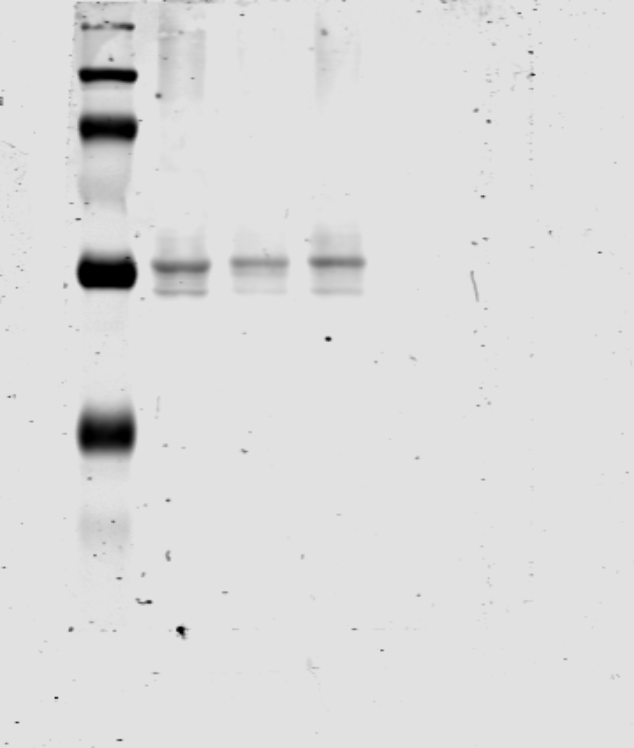

Supplement: Figure 2—source data 2. [file elife-108666-fig2-data2.zip › Figure 2 - Source Data 2. Original files for western blot analysis displayed in Figures 2A, 2C and 2H/ZFT 3HA 3'UTR H7 18h Untreated FAC DFO - CDPK1 loading control stainig gel.tif]

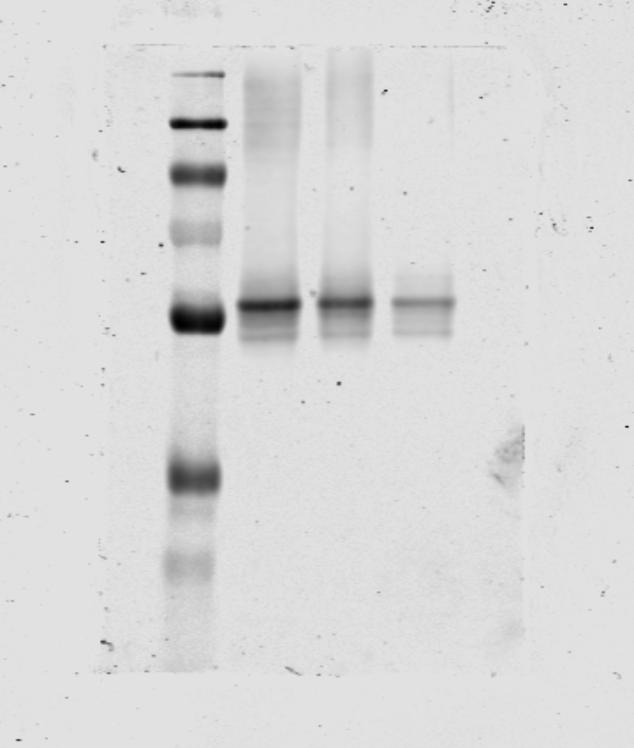

Supplement: Figure 2—source data 2. [file elife-108666-fig2-data2.zip › Figure 2 - Source Data 2. Original files for western blot analysis displayed in Figures 2A, 2C and 2H/ZFT 3HA 3'UTR H7 24h Untreated FAC DFO CDPK1 loading control stainig gel.tif]

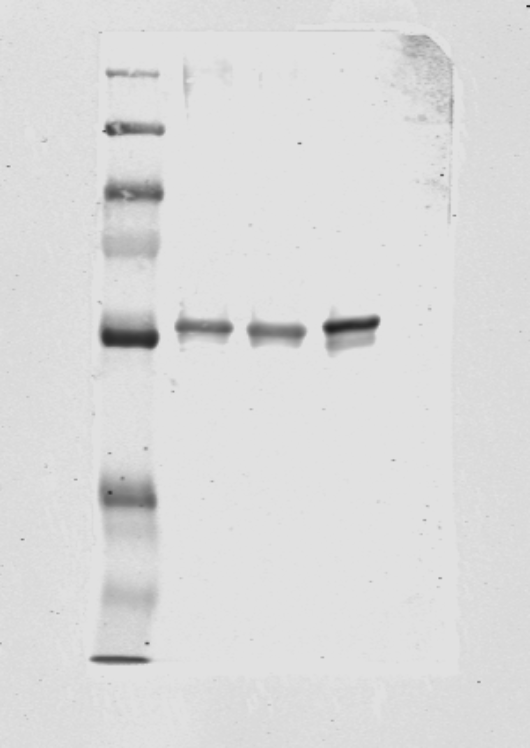

Supplement: Figure 2—source data 2. [file elife-108666-fig2-data2.zip › Figure 2 - Source Data 2. Original files for western blot analysis displayed in Figures 2A, 2C and 2H/RHtdTomato and ZFT-Ty CDPK1 loading control staining.tif]

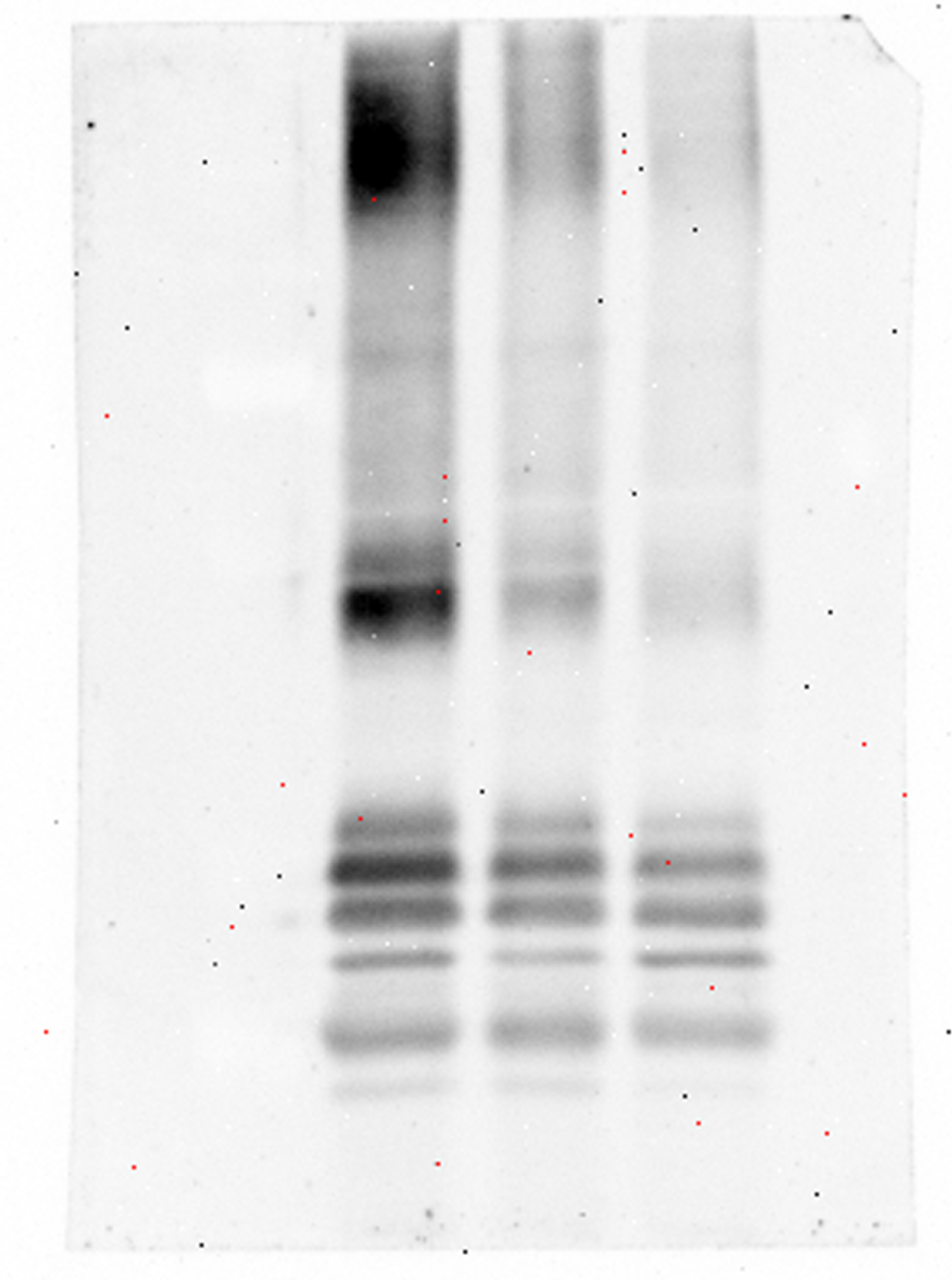

Supplement: Figure 2—source data 2. [file elife-108666-fig2-data2.zip › Figure 2 - Source Data 2. Original files for western blot analysis displayed in Figures 2A, 2C and 2H/ZFT 3HA 3'UTR H7 Untreated_500uM FAC_100uM DFO 24h_17112023_Replicate 4.tif]

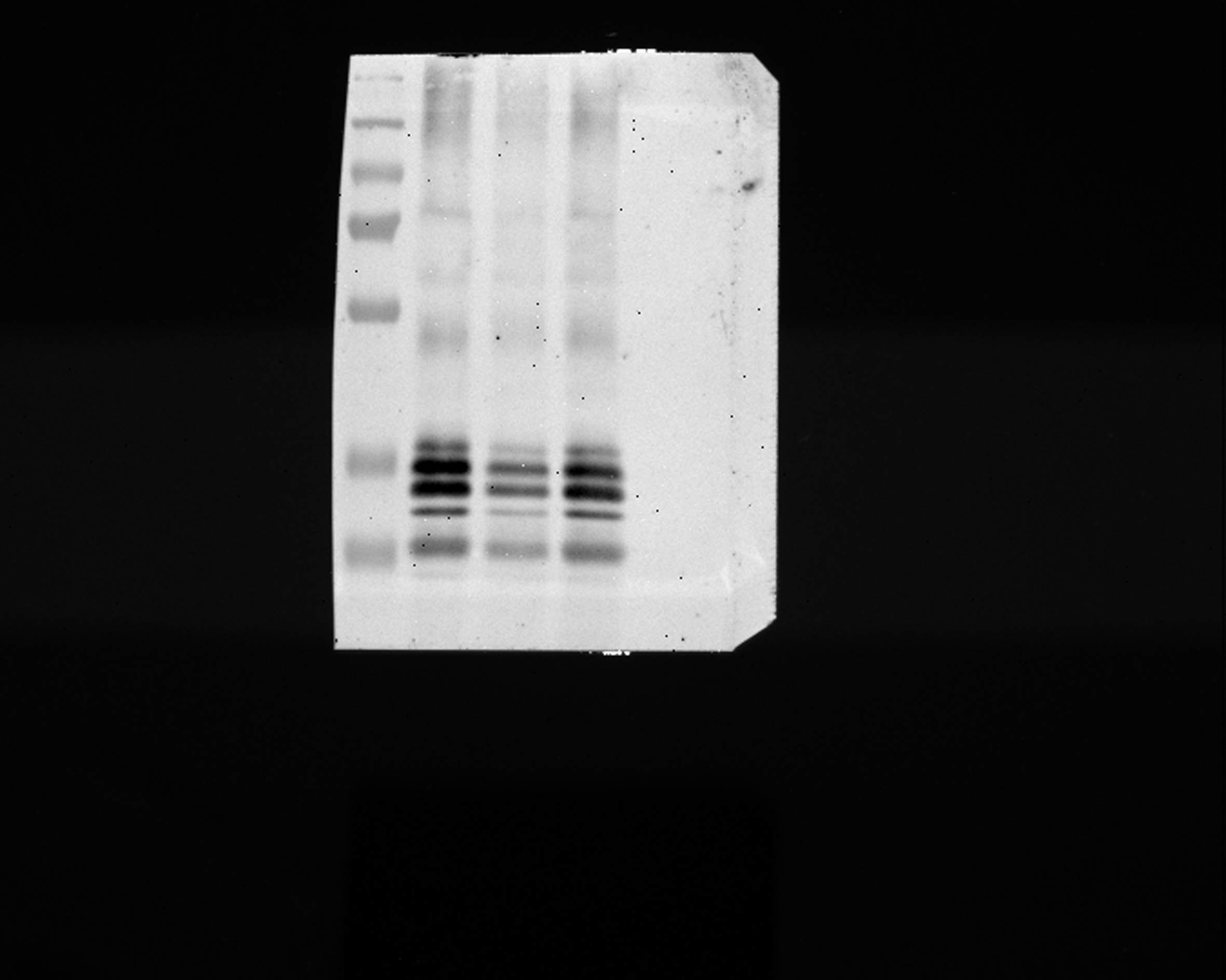

Supplement: Figure 2—source data 2. [file elife-108666-fig2-data2.zip › Figure 2 - Source Data 2. Original files for western blot analysis displayed in Figures 2A, 2C and 2H/CHEMI_11172023_134655.tif]

3E

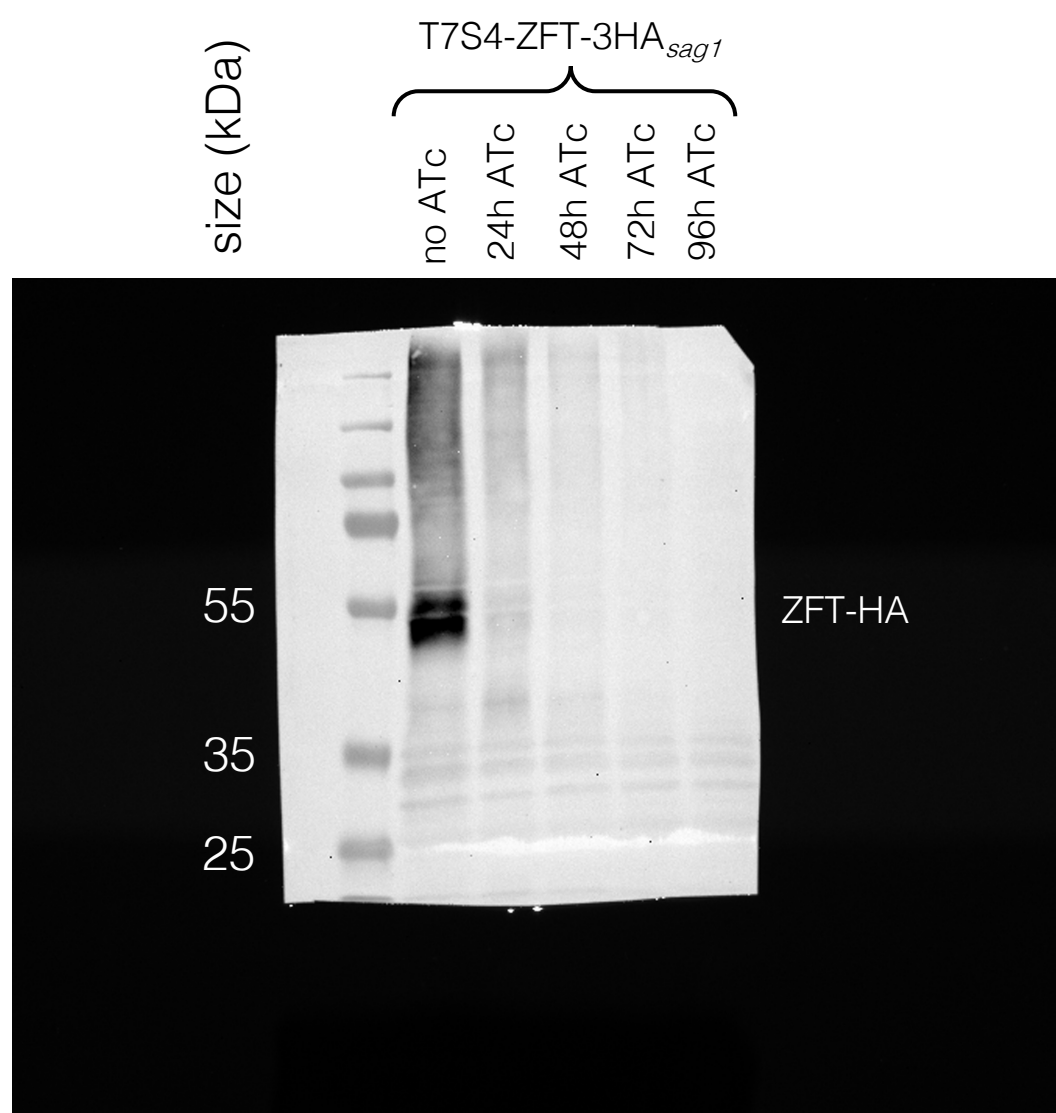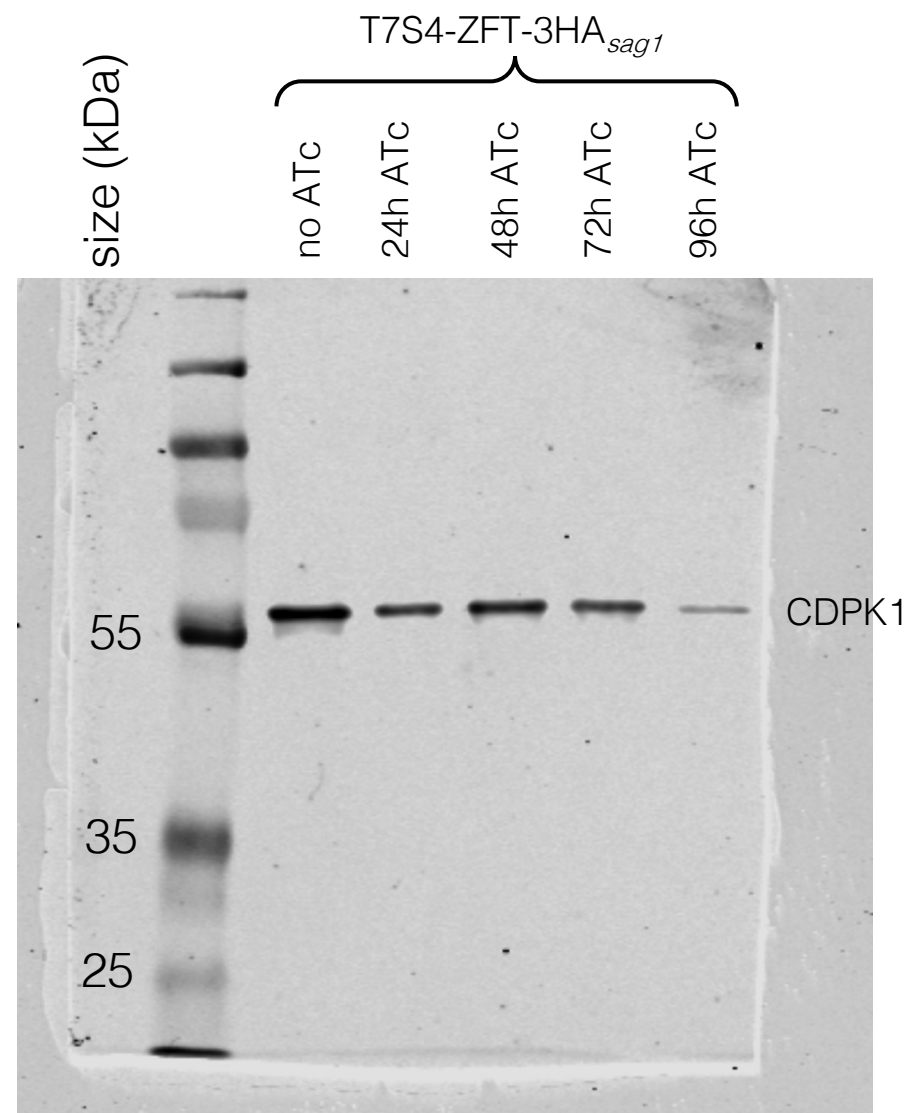

3G

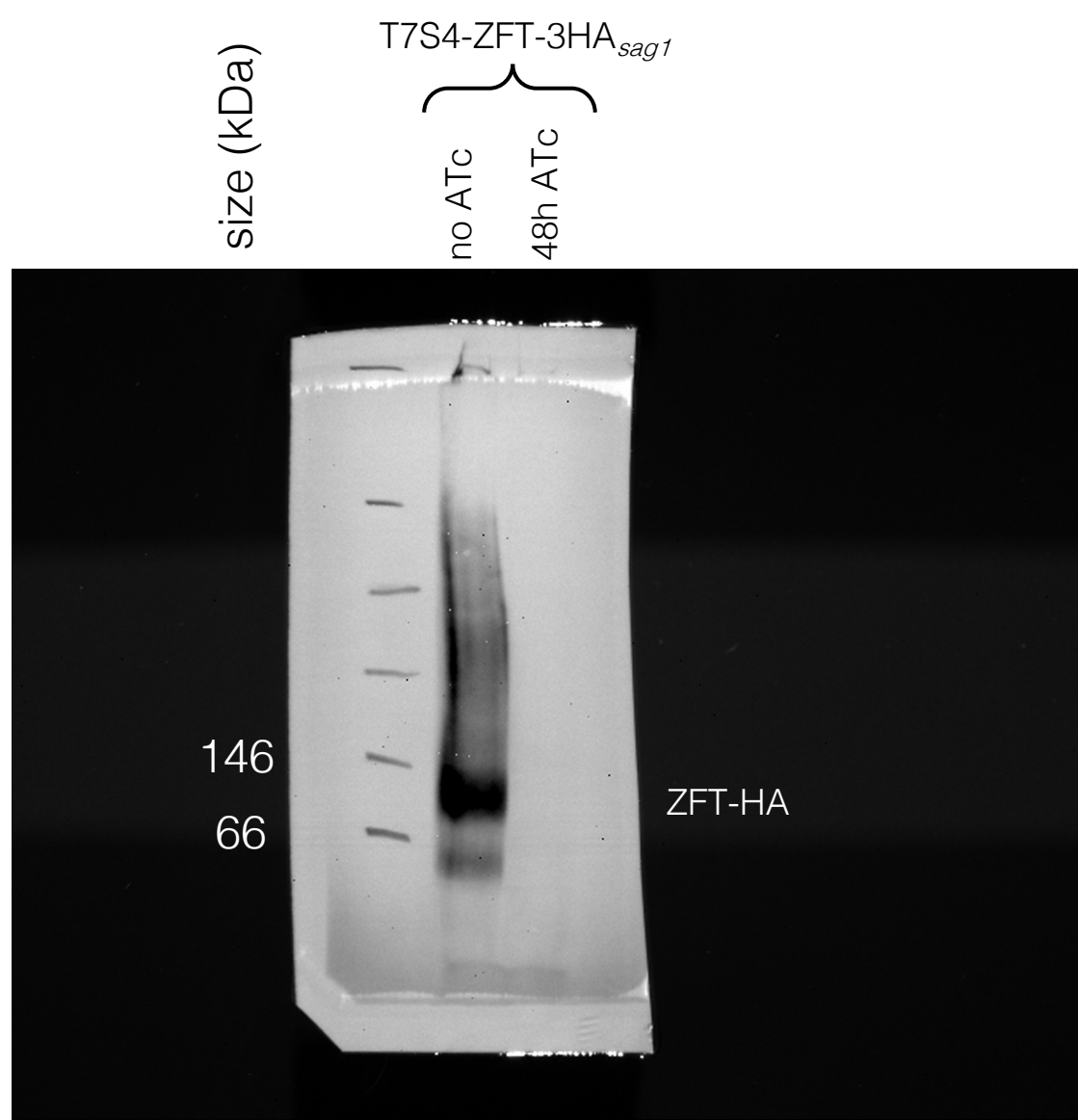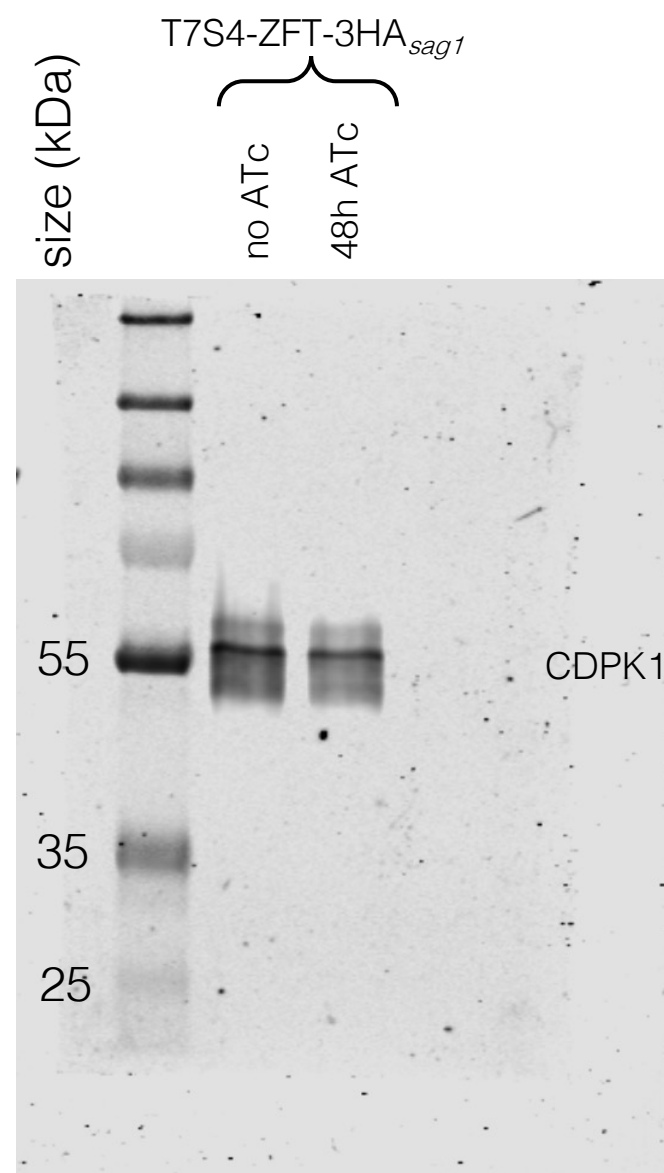

Figure 3, Source Data 1. Original membranes corresponding to Figure 3, panels E and G.

Supplement: Figure 3—source data 1. [file elife-108666-fig3-data1.zip › Figure 3 - Source Data 1. PDF file containing original western blots for Figures 3E and 3G, indicating the relevant bands and conditions/Figure 3_Source Data 1.pdf]

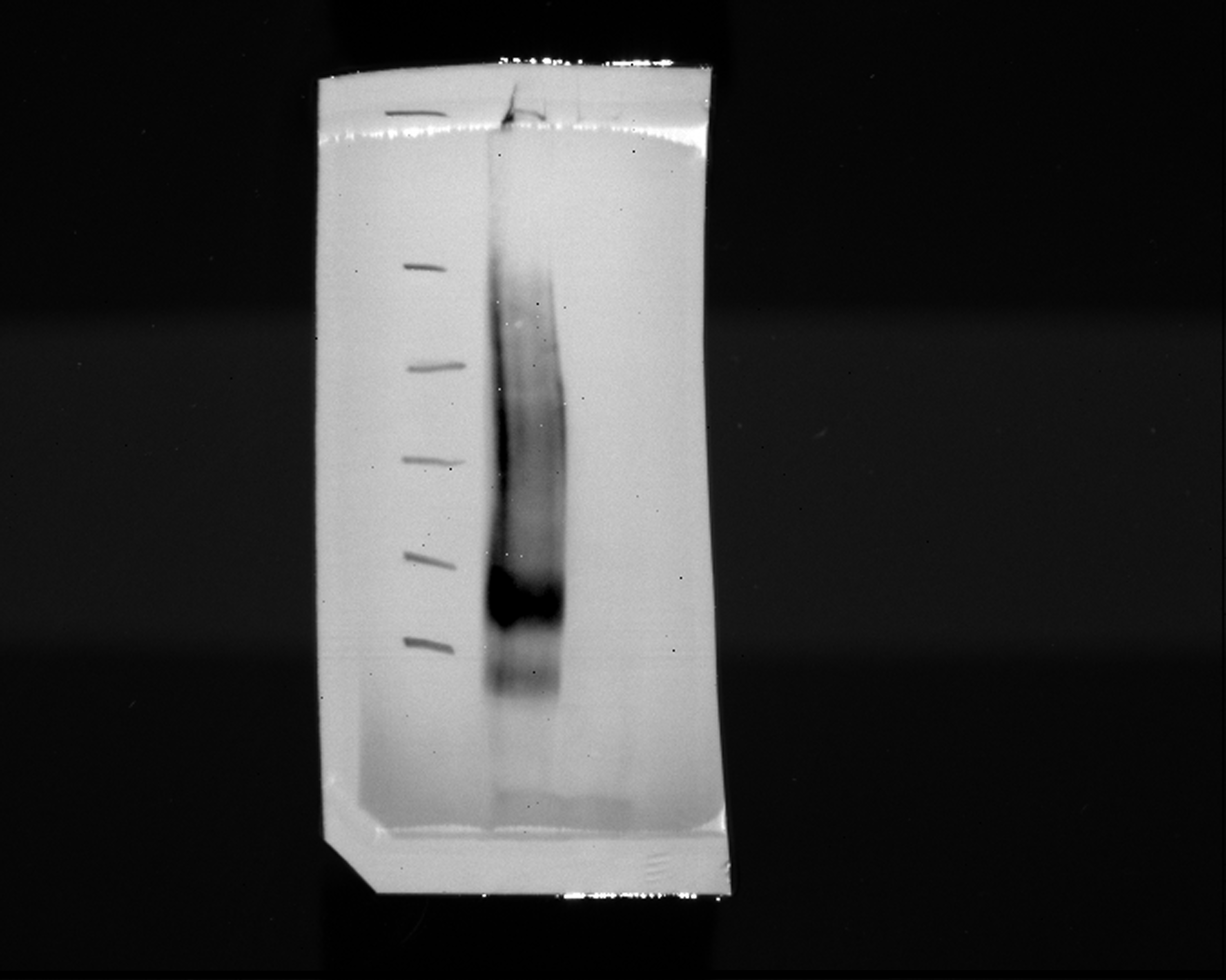

Supplement: Figure 3—source data 2. [file elife-108666-fig3-data2.zip › Figure 3 - Source Data 2. Original files for western blot analysis displayed in Figures 3E and 3G/T7S4 ZFT 3HA E3 no and plus ATc 48h Blue NativePAGE 13092024.tif]

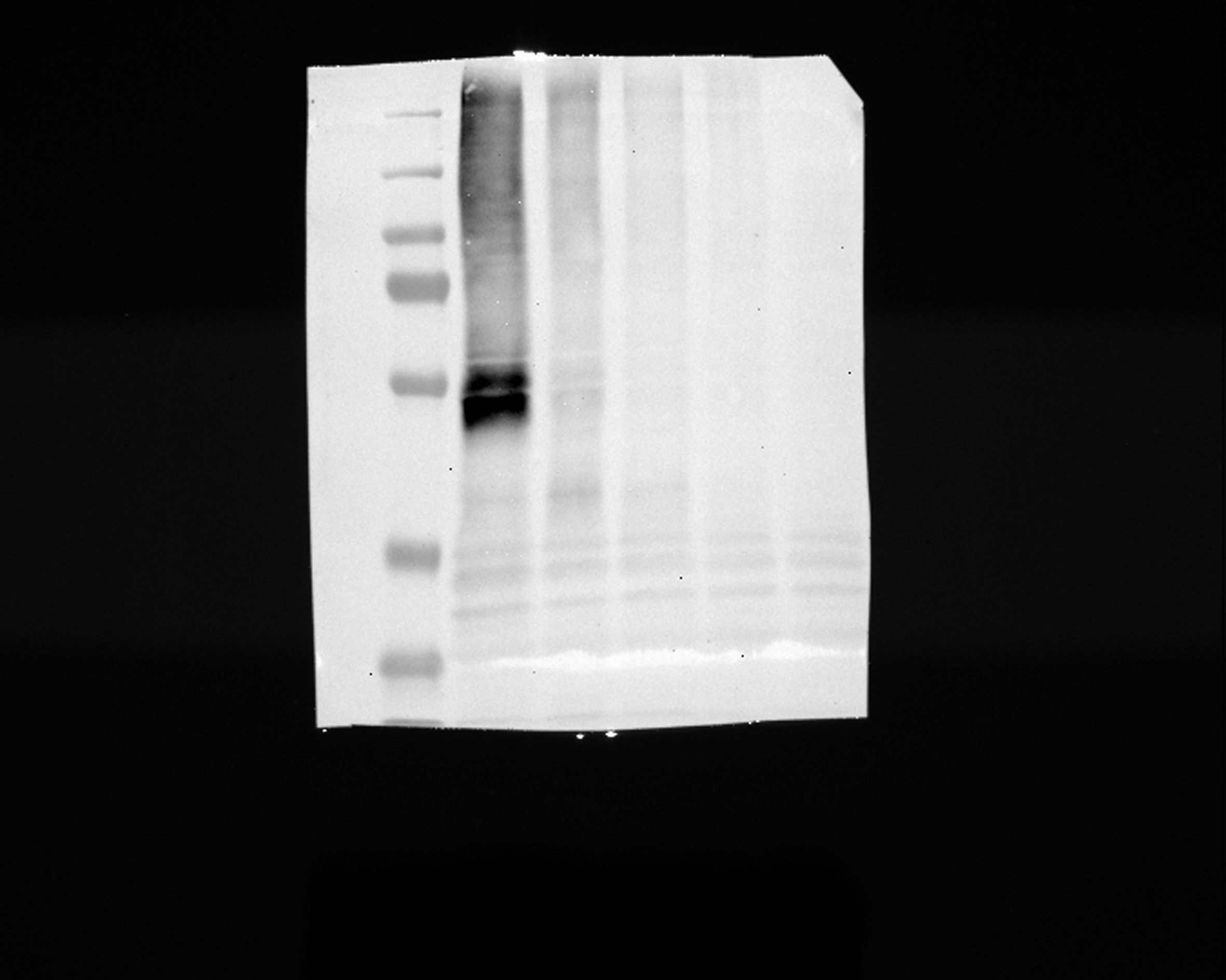

Supplement: Figure 3—source data 2. [file elife-108666-fig3-data2.zip › Figure 3 - Source Data 2. Original files for western blot analysis displayed in Figures 3E and 3G/CHEMI_11302023_130921.tif]

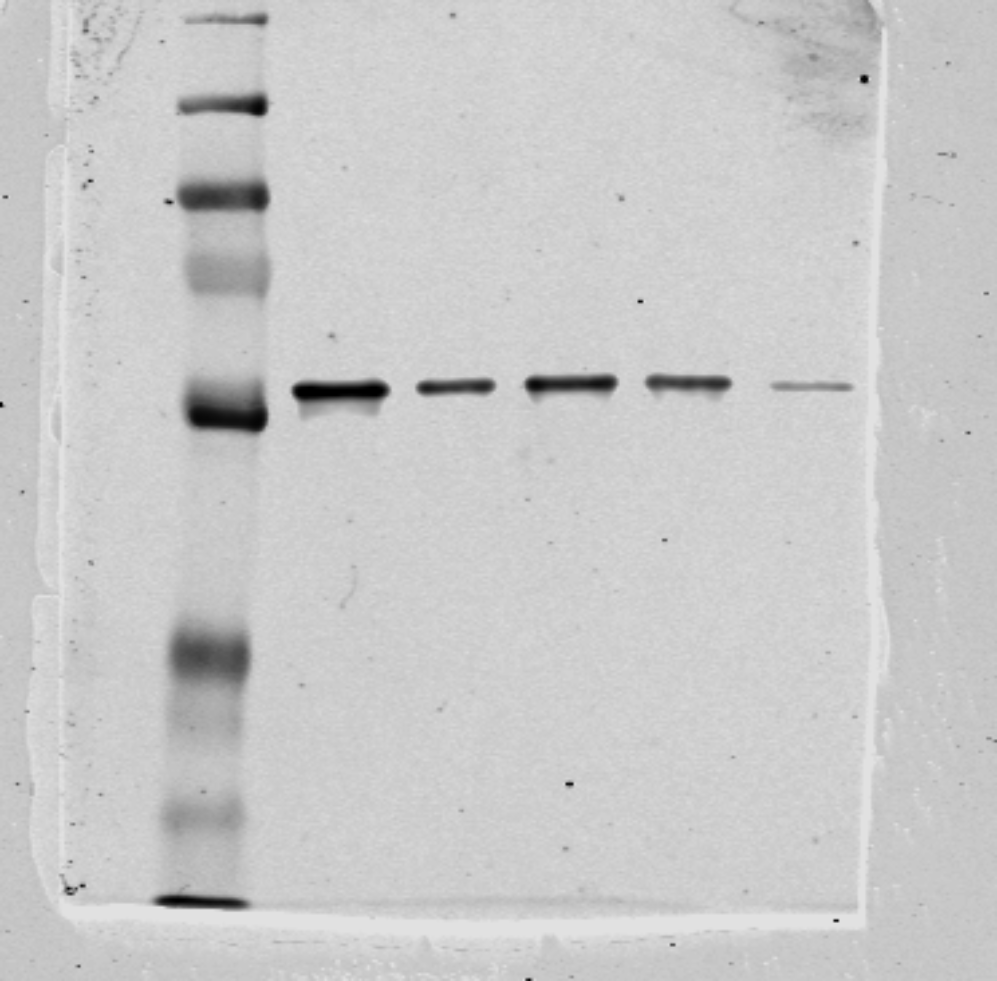

Supplement: Figure 3—source data 2. [file elife-108666-fig3-data2.zip › Figure 3 - Source Data 2. Original files for western blot analysis displayed in Figures 3E and 3G/T7S4 ZFT 3HA no and plus ATc 24h 48h 72h 96h CDPK1 loading control Replicate 2.tif]

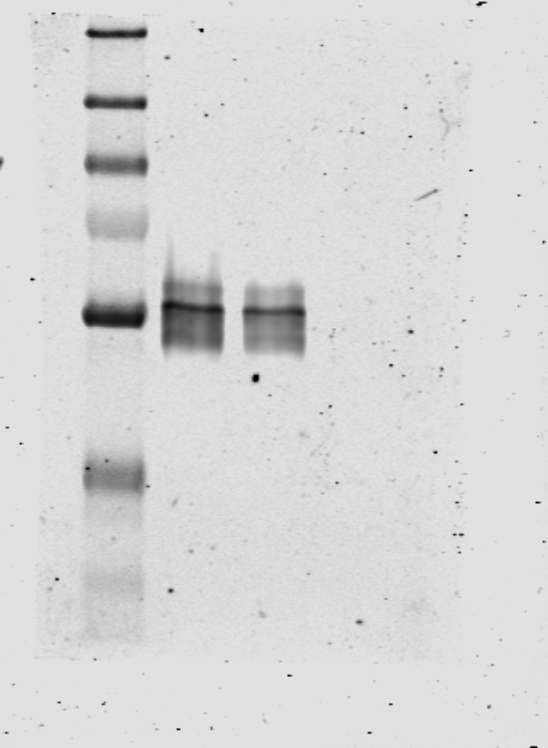

Supplement: Figure 3—source data 2. [file elife-108666-fig3-data2.zip › Figure 3 - Source Data 2. Original files for western blot analysis displayed in Figures 3E and 3G/T7S4 ZFT 3HA E3 no and plus ATc 48h CDPK1 Loading Control for Blue NativePAGE.tif]

3C

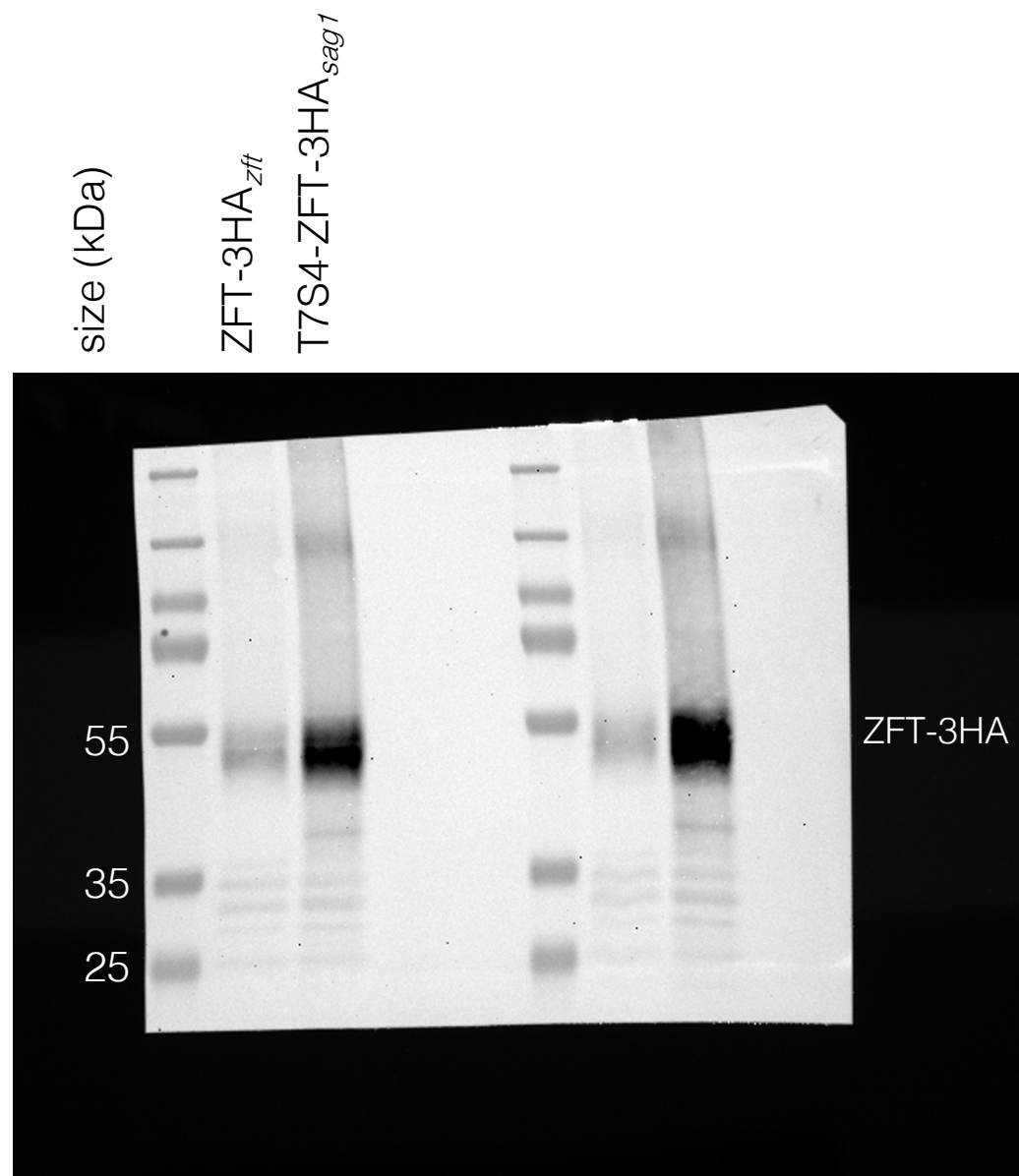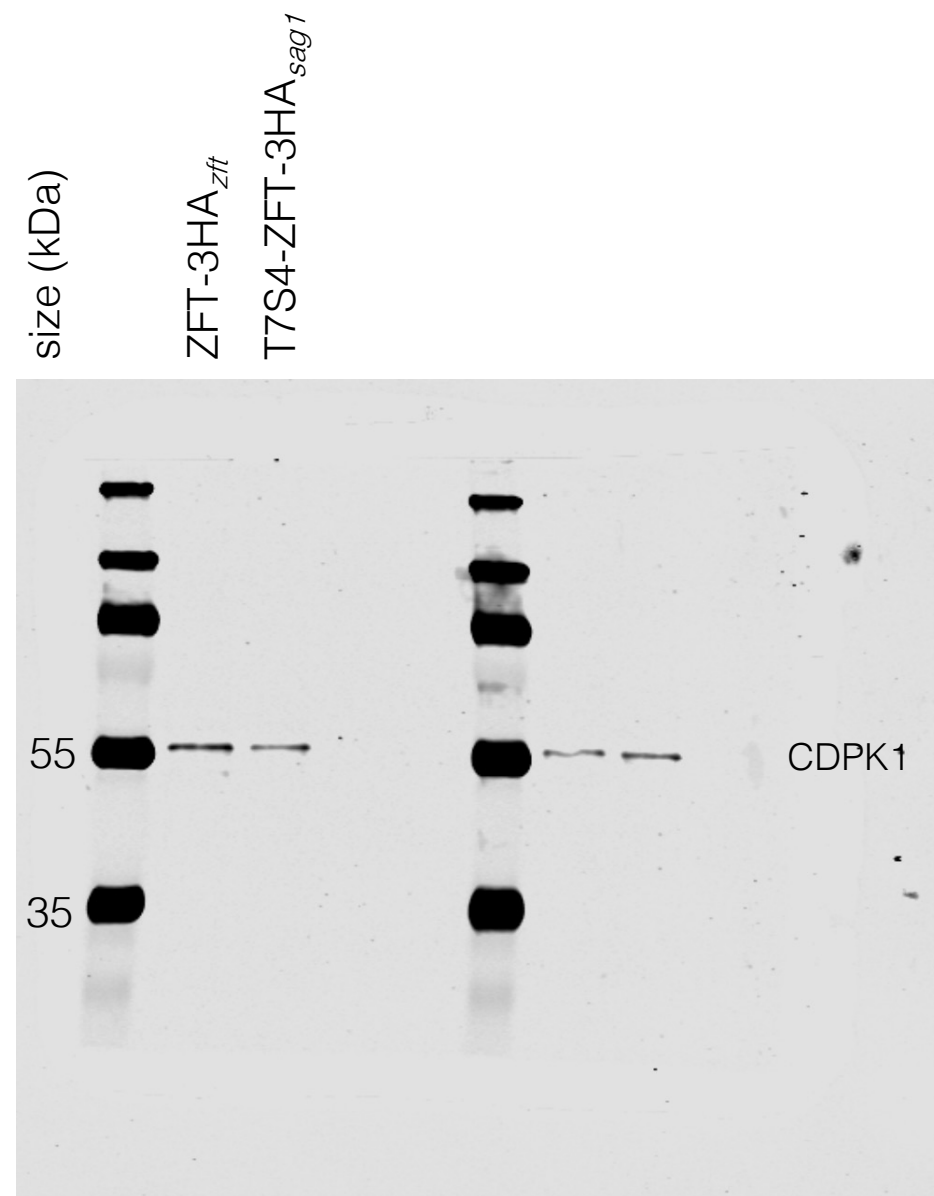

3E

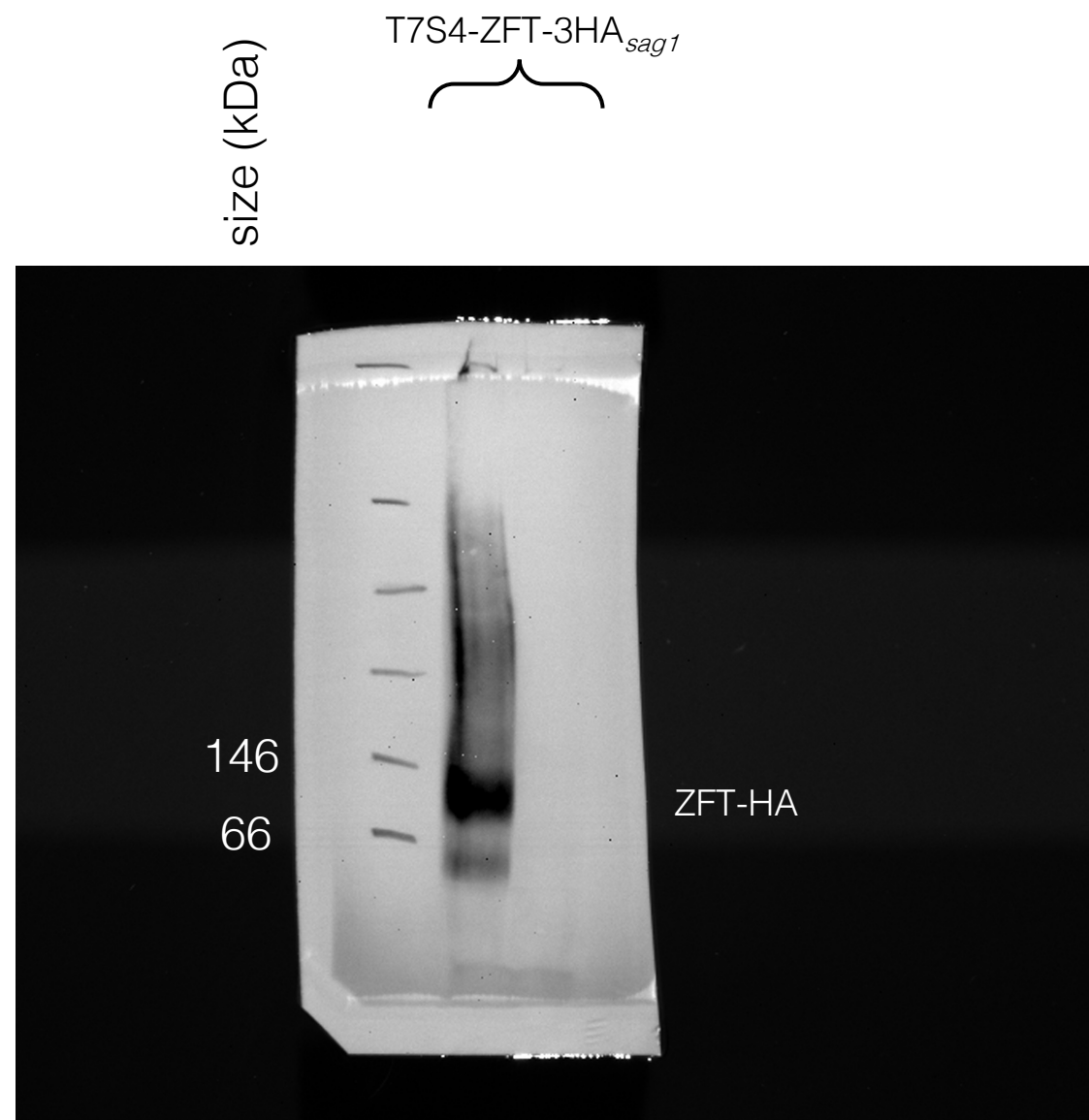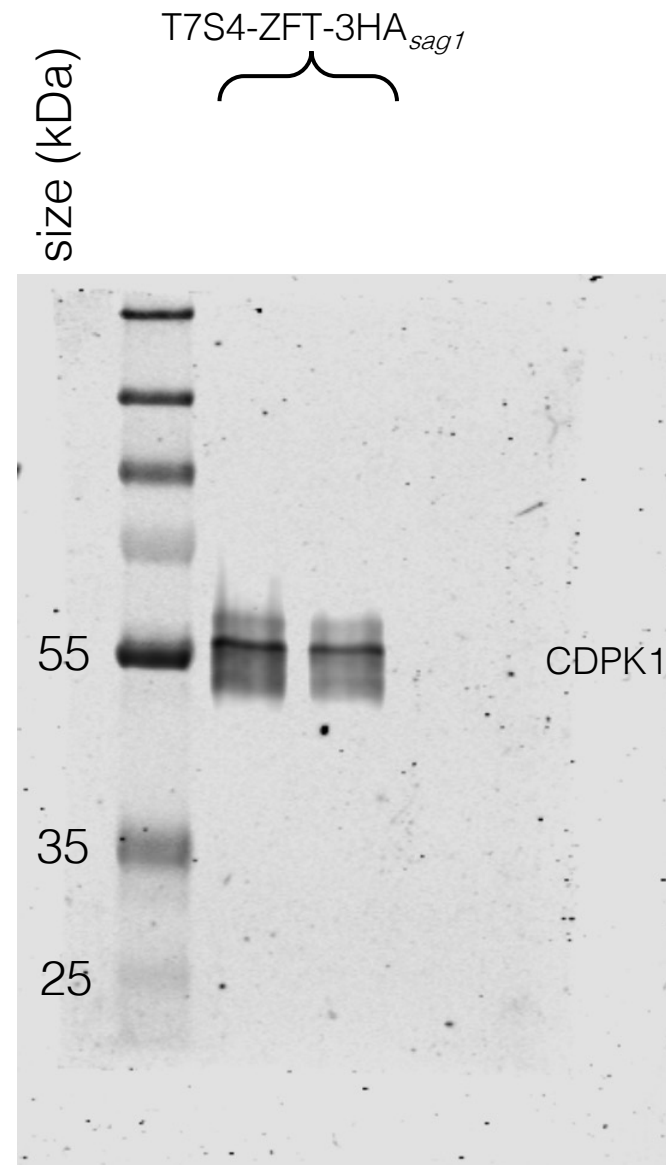

Figure S3, Source Data 1. Original membranes corresponding to Figure S3, panels C and E.

Supplement: Figure 3—figure supplement 1—source data 1. [file elife-108666-fig3-figsupp1-data1.zip › Figure S3 - Source Data 1. PDF file containing original western blots for Figures S3C and S3E, indicating the relevant bands and conditions/Figure S3_Source Data 1.pdf]

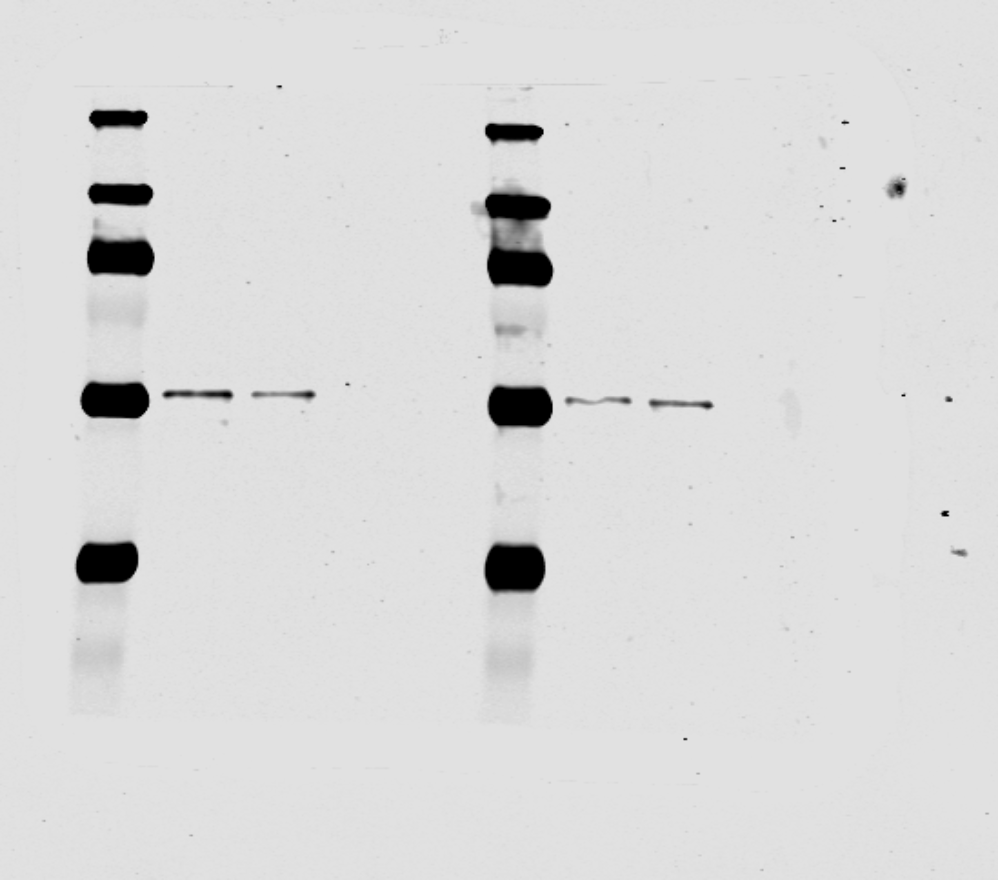

Supplement: Figure 3—figure supplement 1—source data 2. [file elife-108666-fig3-figsupp1-data2.zip › Figure S3 - Source Data 2. Original files for western blot analysis displayed in Figures S3C and S3E/ZFT 3HA 3UTR and T7S4 ZFT 3HA Replicates 1 and 2 - Overexpression Westerns CDPK1 loading control staining.tif]

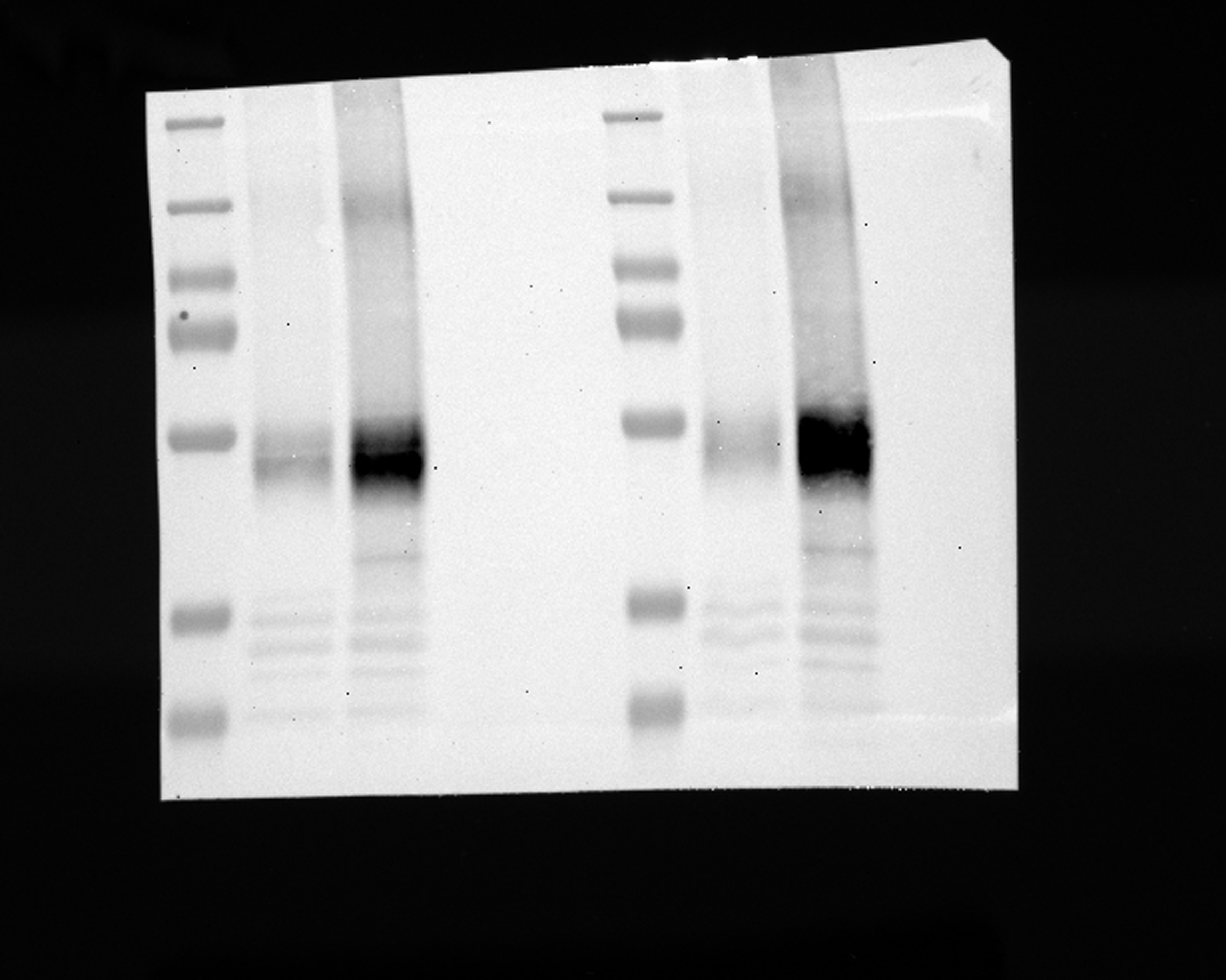

Supplement: Figure 3—figure supplement 1—source data 2. [file elife-108666-fig3-figsupp1-data2.zip › Figure S3 - Source Data 2. Original files for western blot analysis displayed in Figures S3C and S3E/CHEMI_05132025_112639.tif]

4B

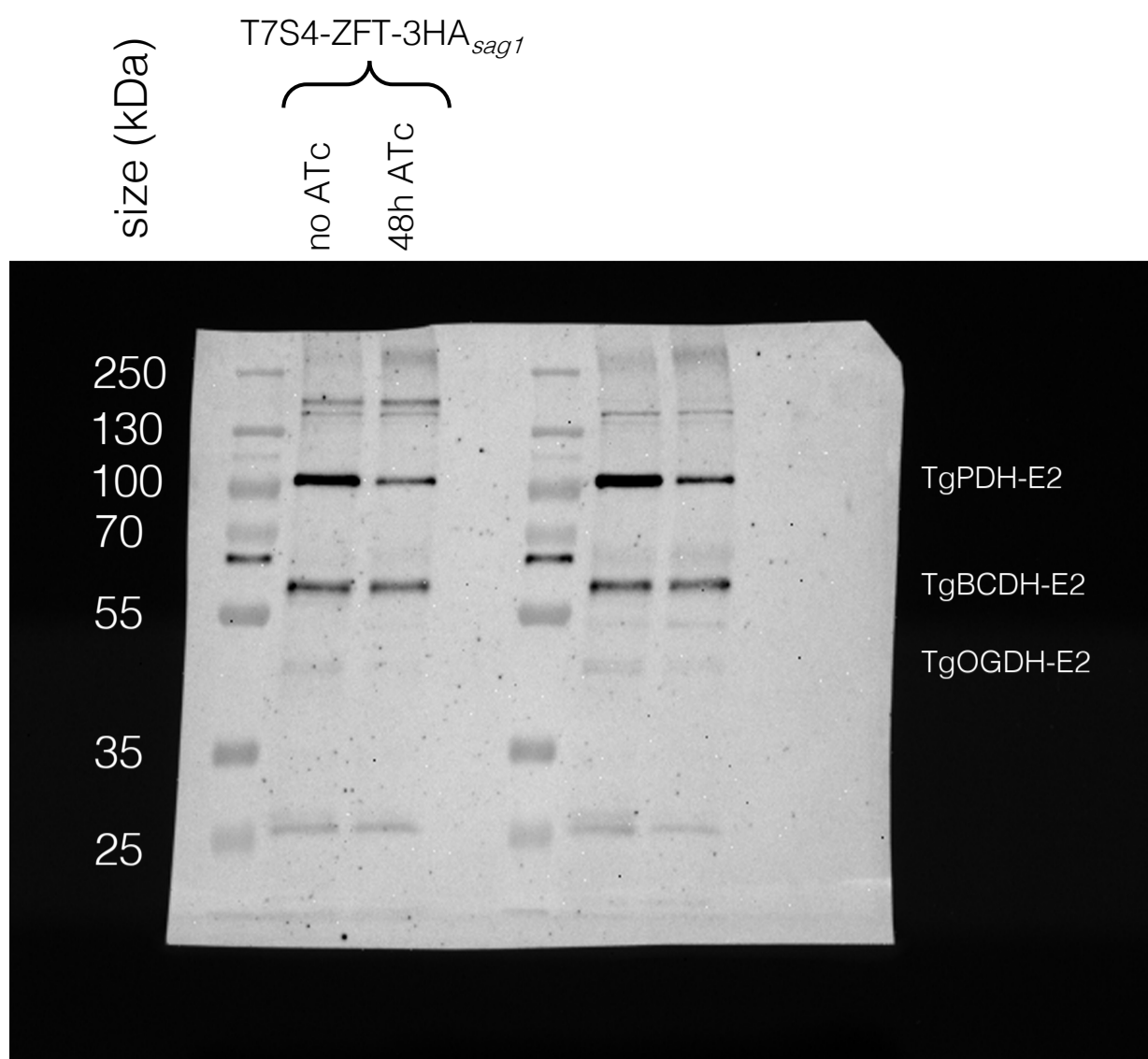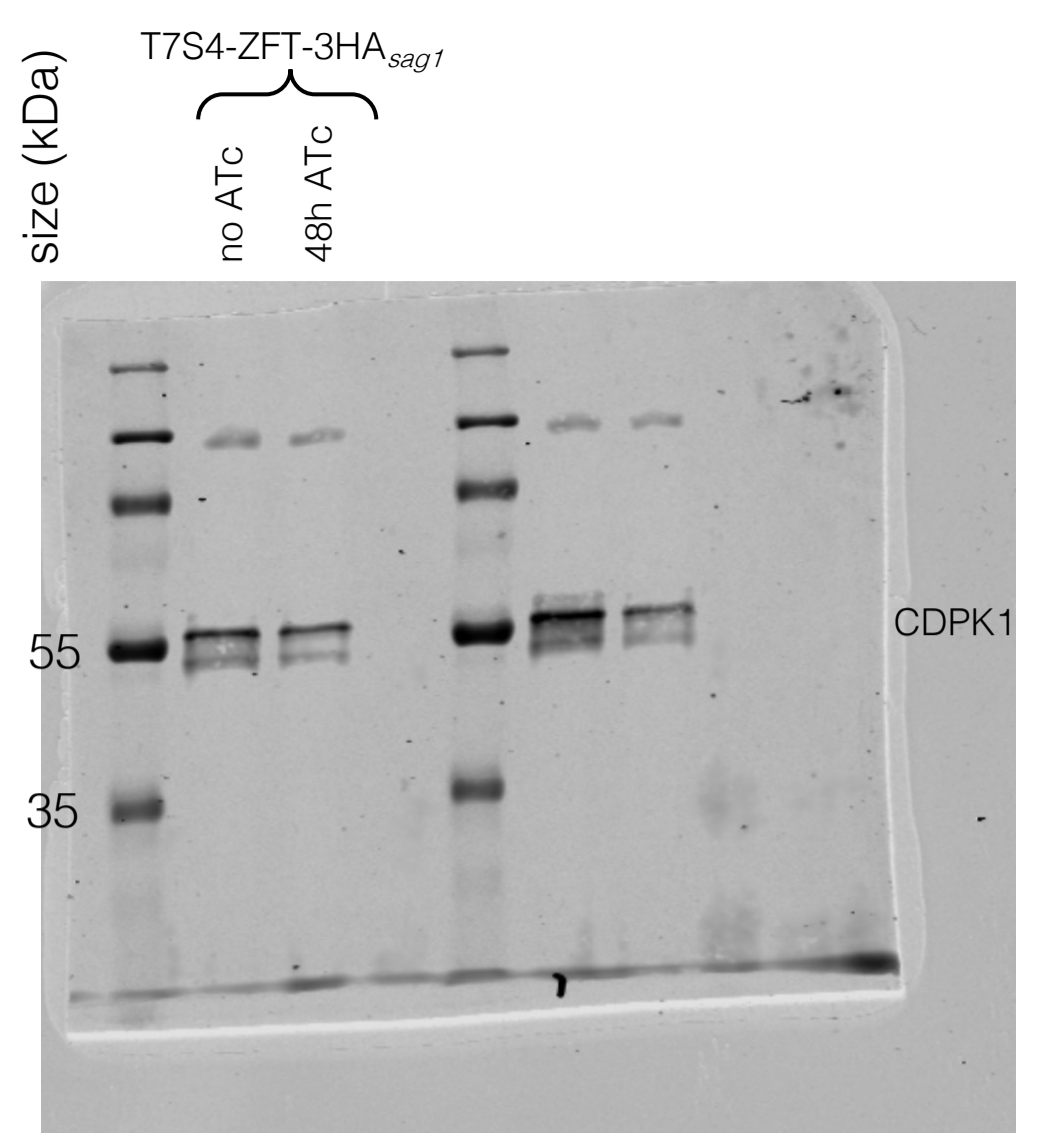

4K

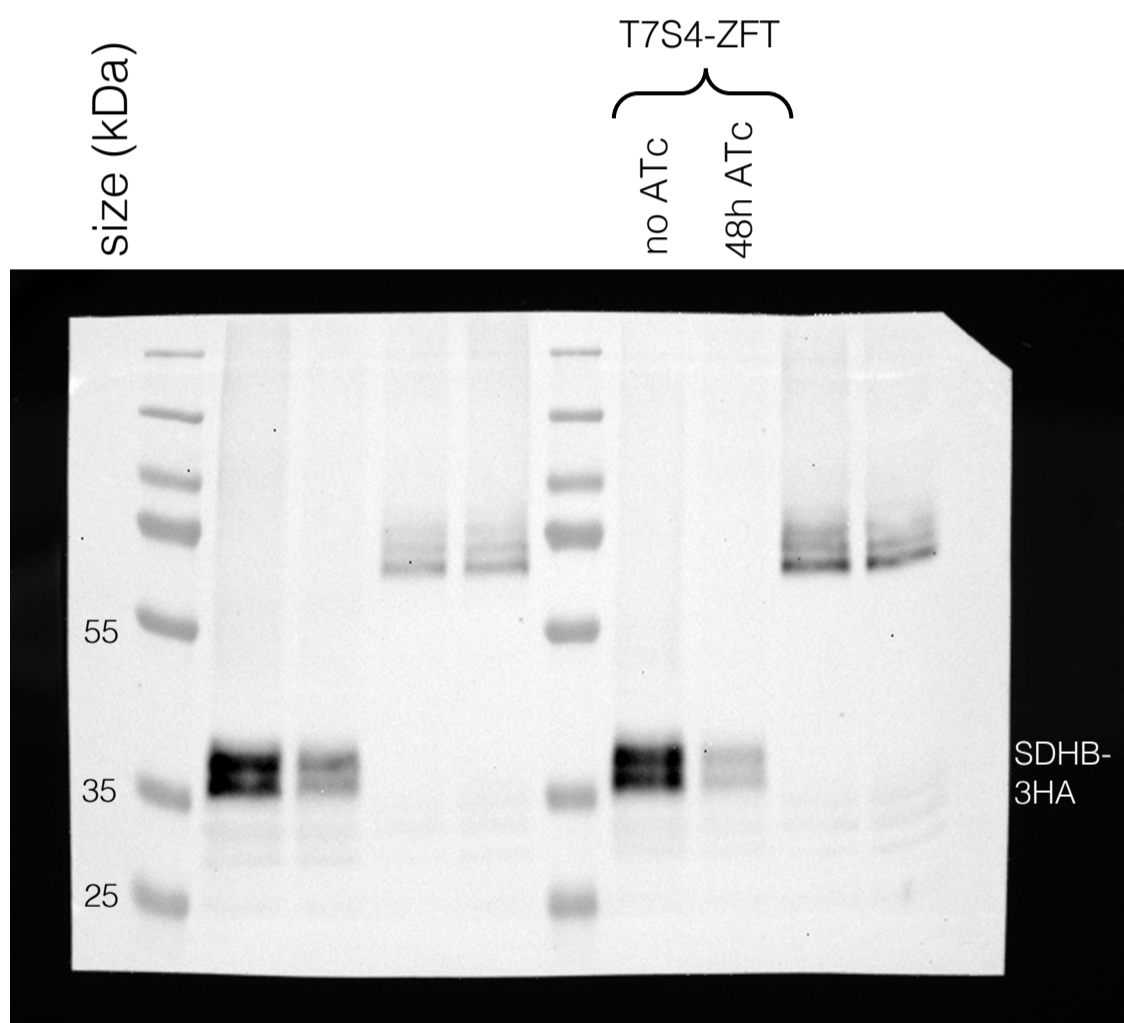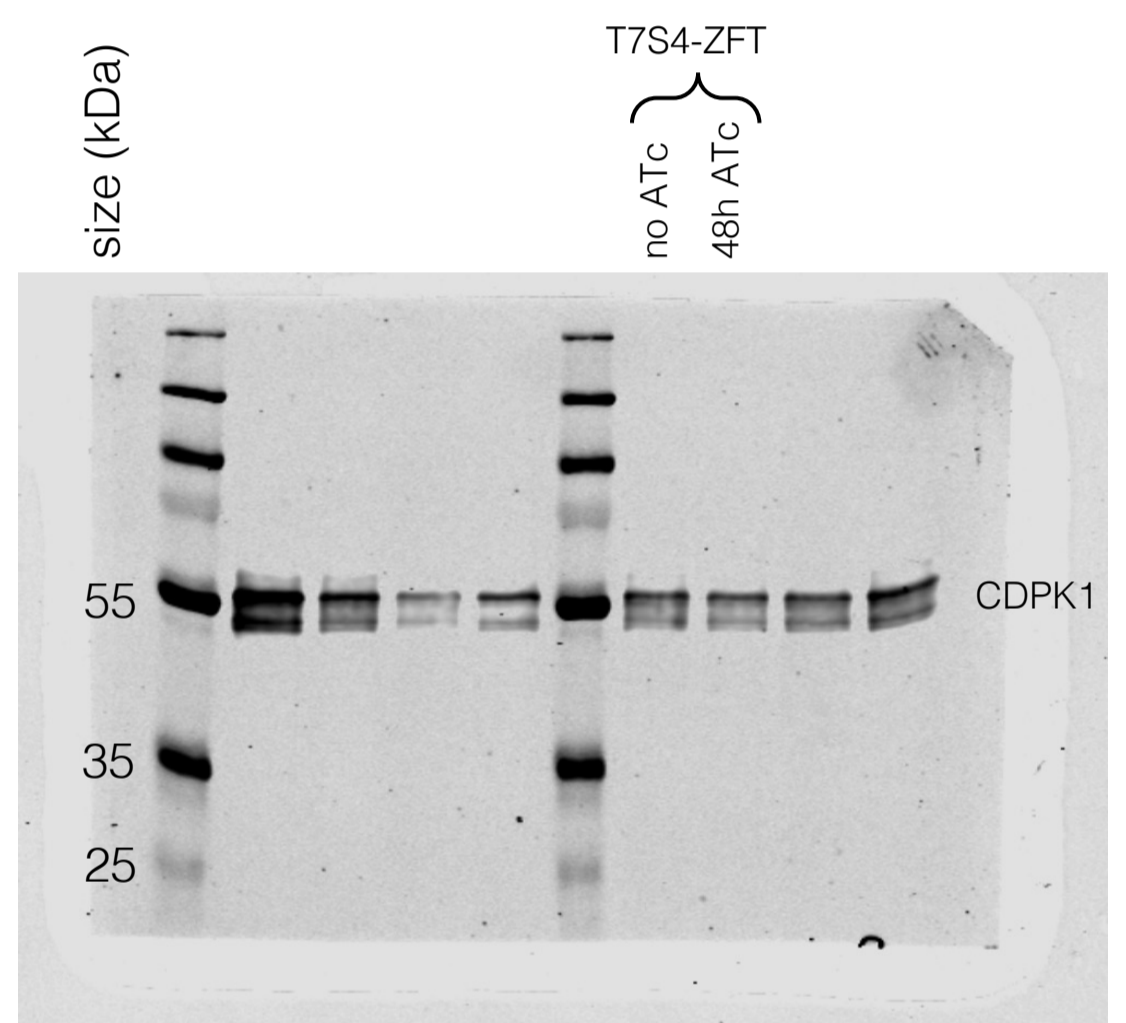

4M

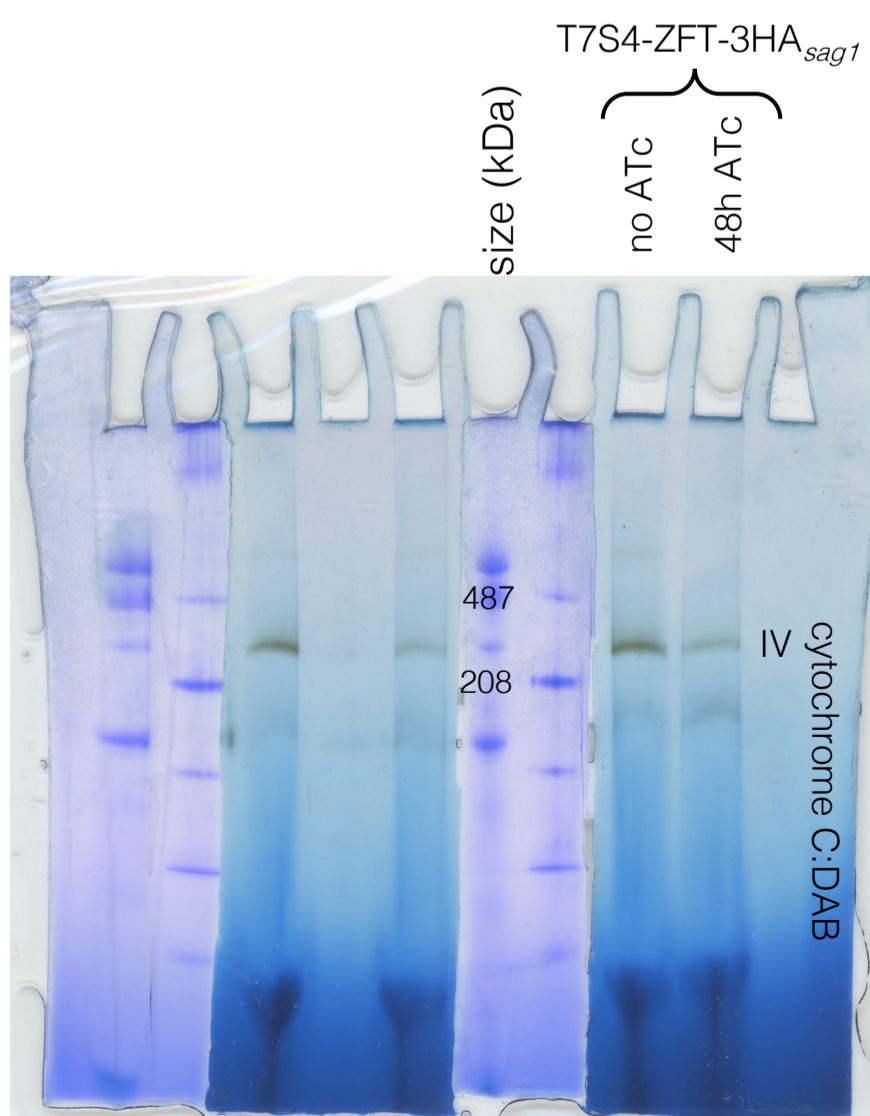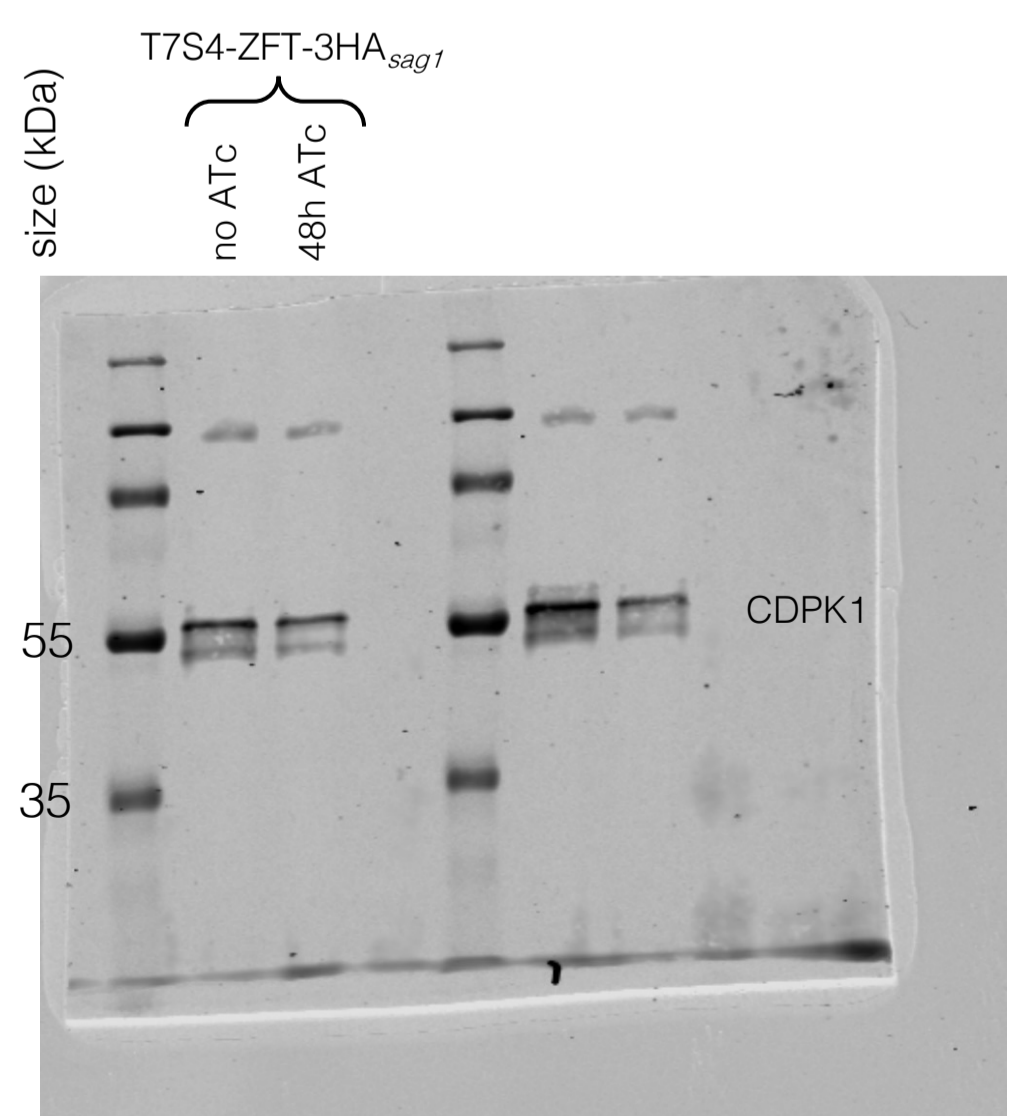

Figure 4, Source Data 1. Original membranes corresponding to Figure 4, panels B, K and M.

Supplement: Figure 4—source data 1. [file elife-108666-fig4-data1.zip › Figure 4 - Source Data 1. PDF file containing original western blots for Figures 4B, 4K and 4M indicating the relevant bands and conditions/Figure 4_Source Data 1.pdf]

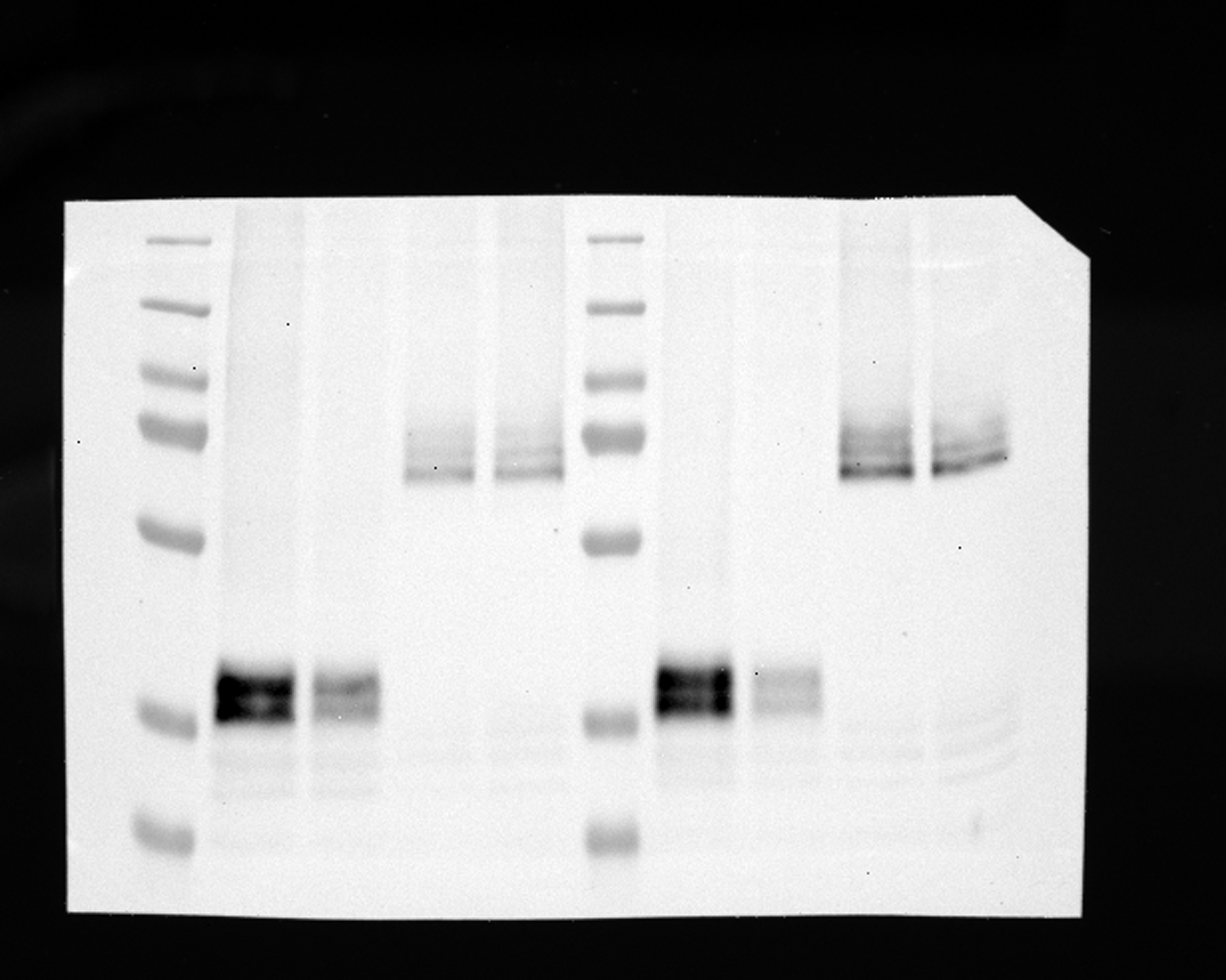

Supplement: Figure 4—source data 2. [file elife-108666-fig4-data2.zip › Figure 4 - Source Data 2. Original files for western blot analysis displayed in Figures 4B, 4K and 4M/CHEMI_11072024_150443.tif]

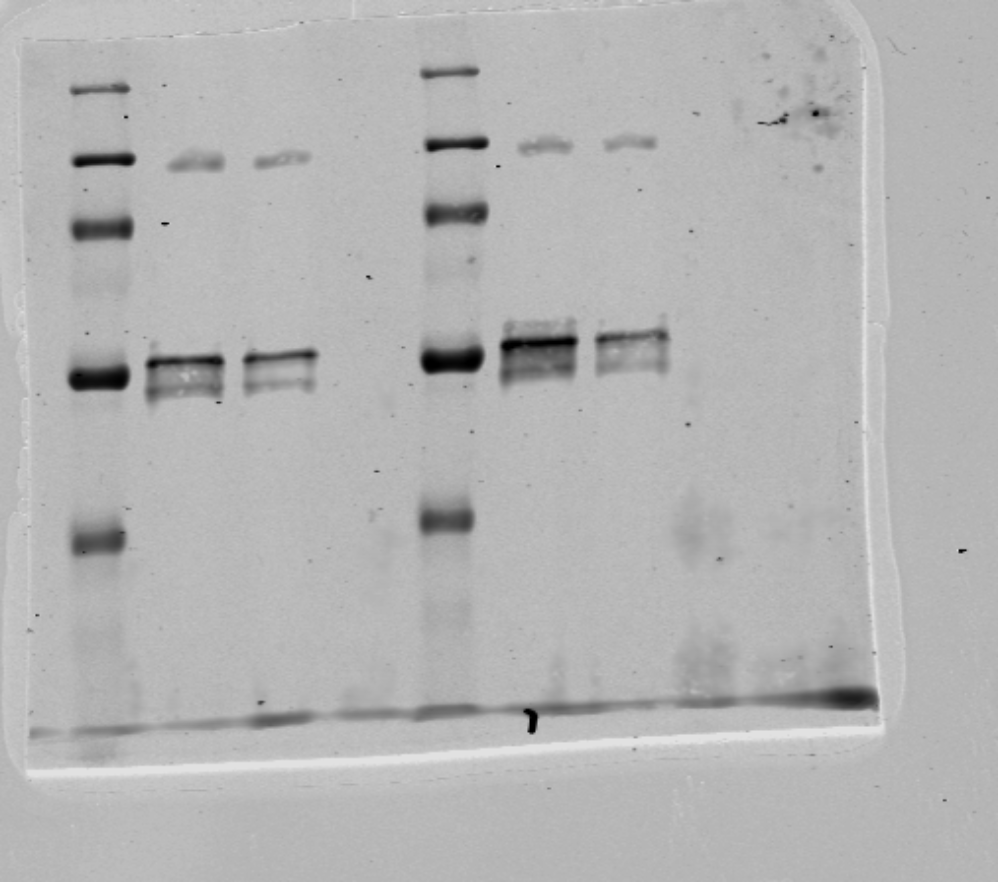

Supplement: Figure 4—source data 2. [file elife-108666-fig4-data2.zip › Figure 4 - Source Data 2. Original files for western blot analysis displayed in Figures 4B, 4K and 4M/T7S4 ZFT 3HA no and plus ATc 48h Replicates 3 and 4 loading control_CDPK1 staining for complex IV activity assay.tif]

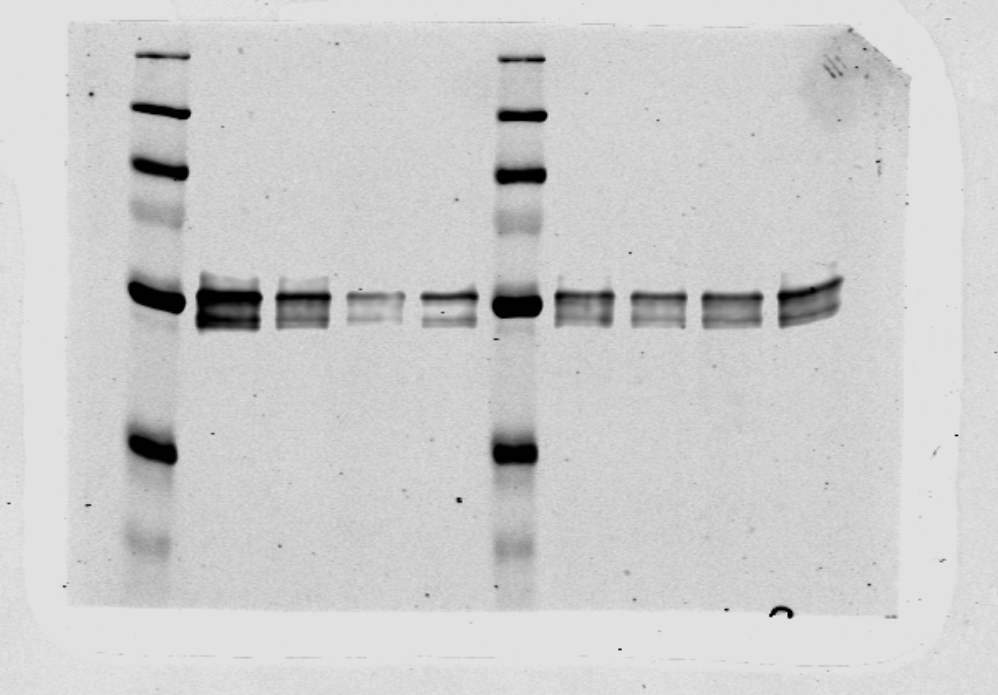

Supplement: Figure 4—source data 2. [file elife-108666-fig4-data2.zip › Figure 4 - Source Data 2. Original files for western blot analysis displayed in Figures 4B, 4K and 4M/T7S4 ZFT SDHB-HA and ABCE1-HA no and plus ATc 48h Replicates 1 and 2 CDPK1 Loading Control Staining.tif]

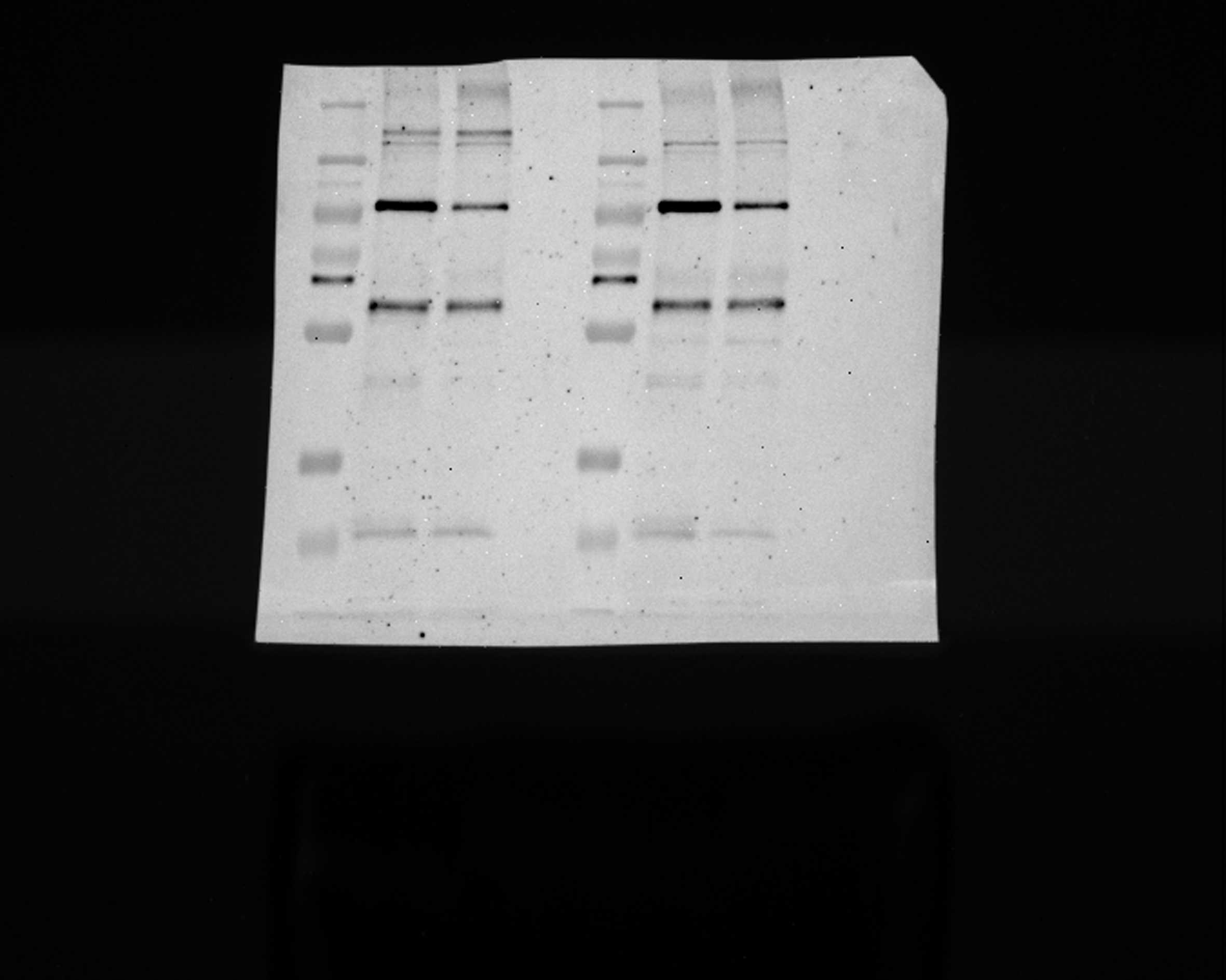

Supplement: Figure 4—source data 2. [file elife-108666-fig4-data2.zip › Figure 4 - Source Data 2. Original files for western blot analysis displayed in Figures 4B, 4K and 4M/CHEMI_05212025_142906.tif]

S4A

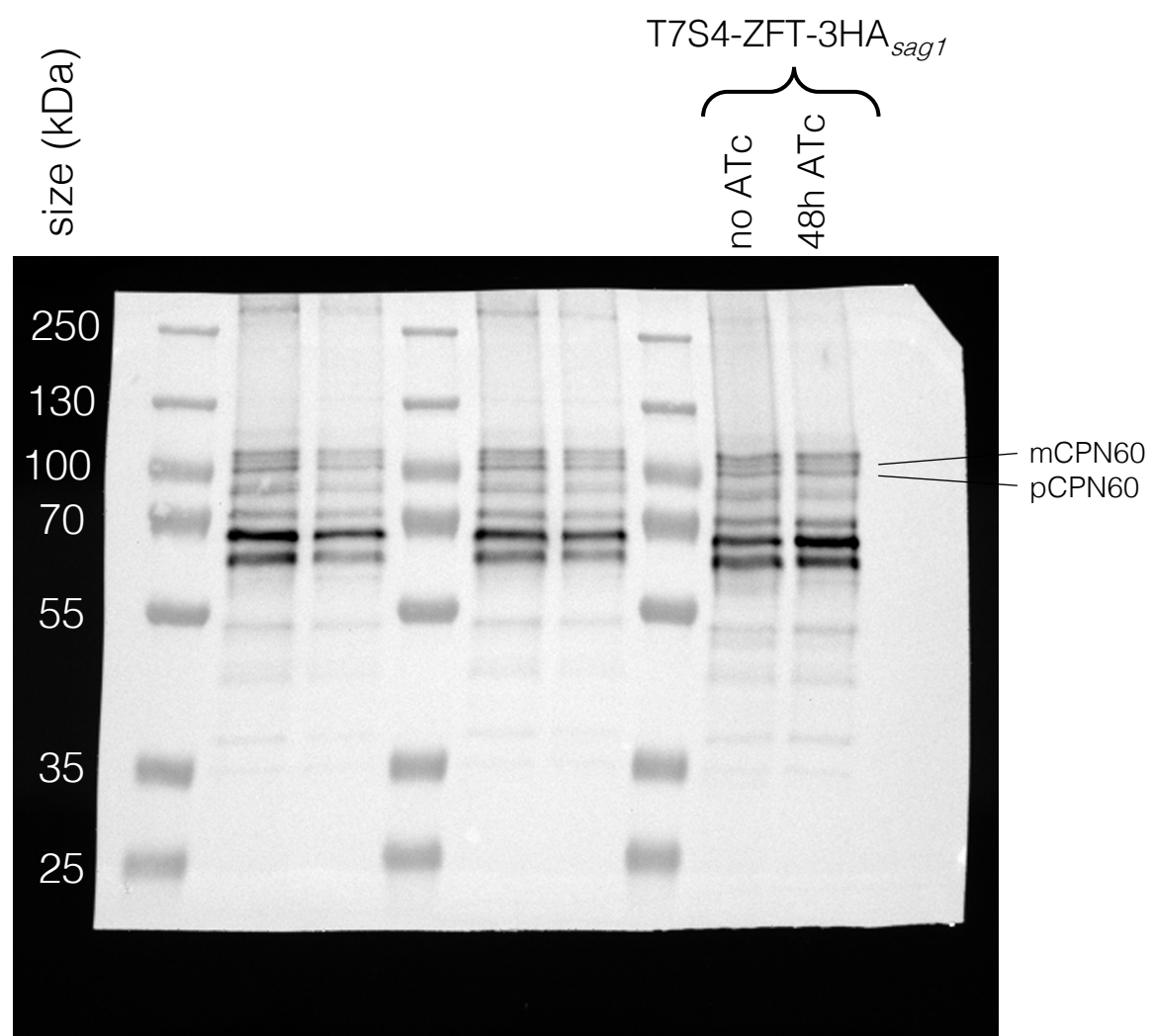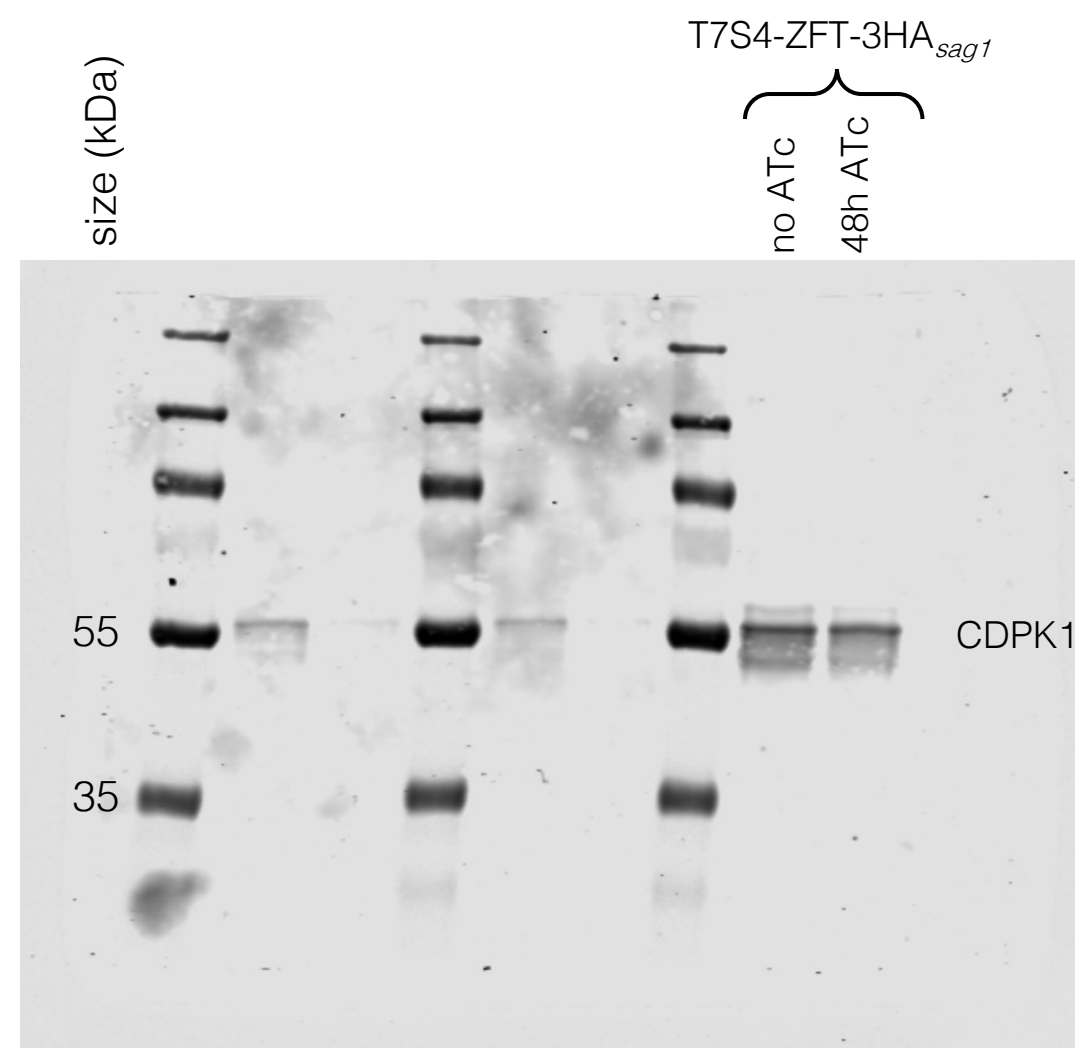

S4J

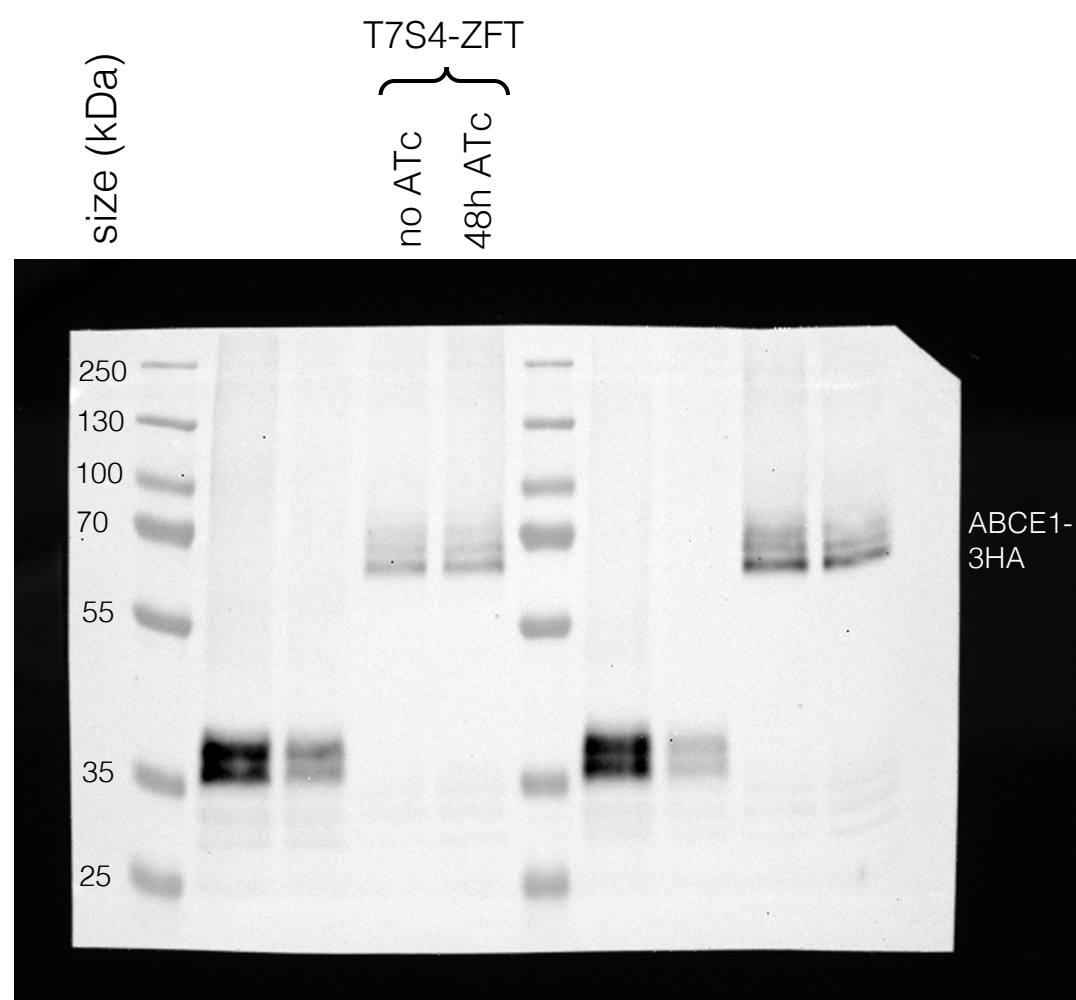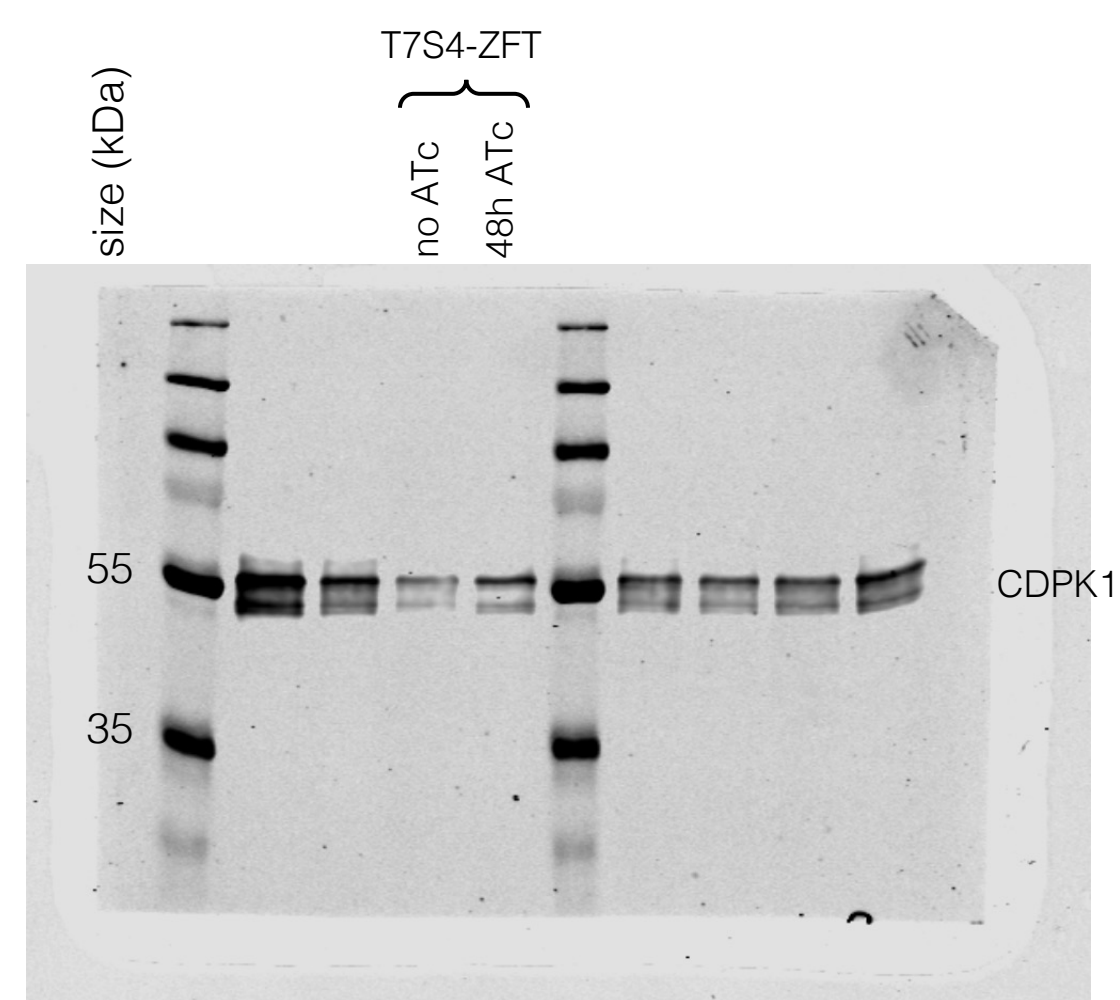

Figure S4, Source Data 1. Original membranes corresponding to Figure S4, panels A and J.

Supplement: Figure 4—figure supplement 1—source data 1. [file elife-108666-fig4-figsupp1-data1.zip › Figure S4 - Source Data 1. PDF file containing original western blots for Figures S4A and S4J, indicating the relevant bands and conditions/Figure S4_Source Data 1.pdf]

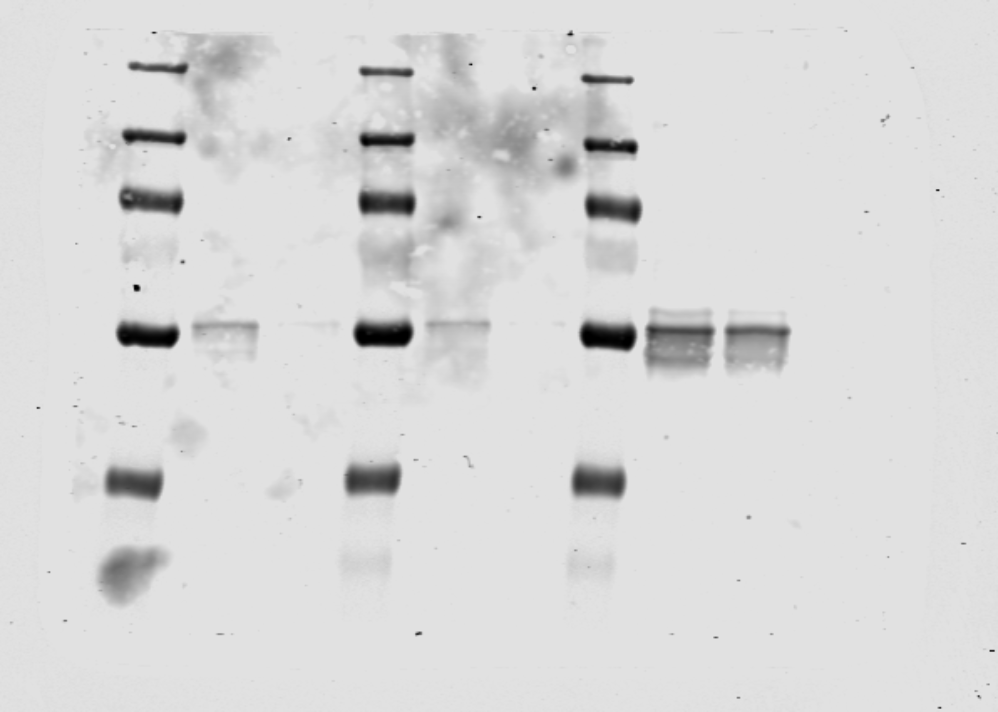

Supplement: Figure 4—figure supplement 1—source data 2. [file elife-108666-fig4-figsupp1-data2.zip › Figure S4 - Source Data 2. Original files for western blot analysis displayed in Figures S4A and S4J/T7S4 ZFT 3HA no and plus ATc 48h Replicates 1 2 and 3 - CPN60 apicoplast western - CDPK1 loading control staining.tif]

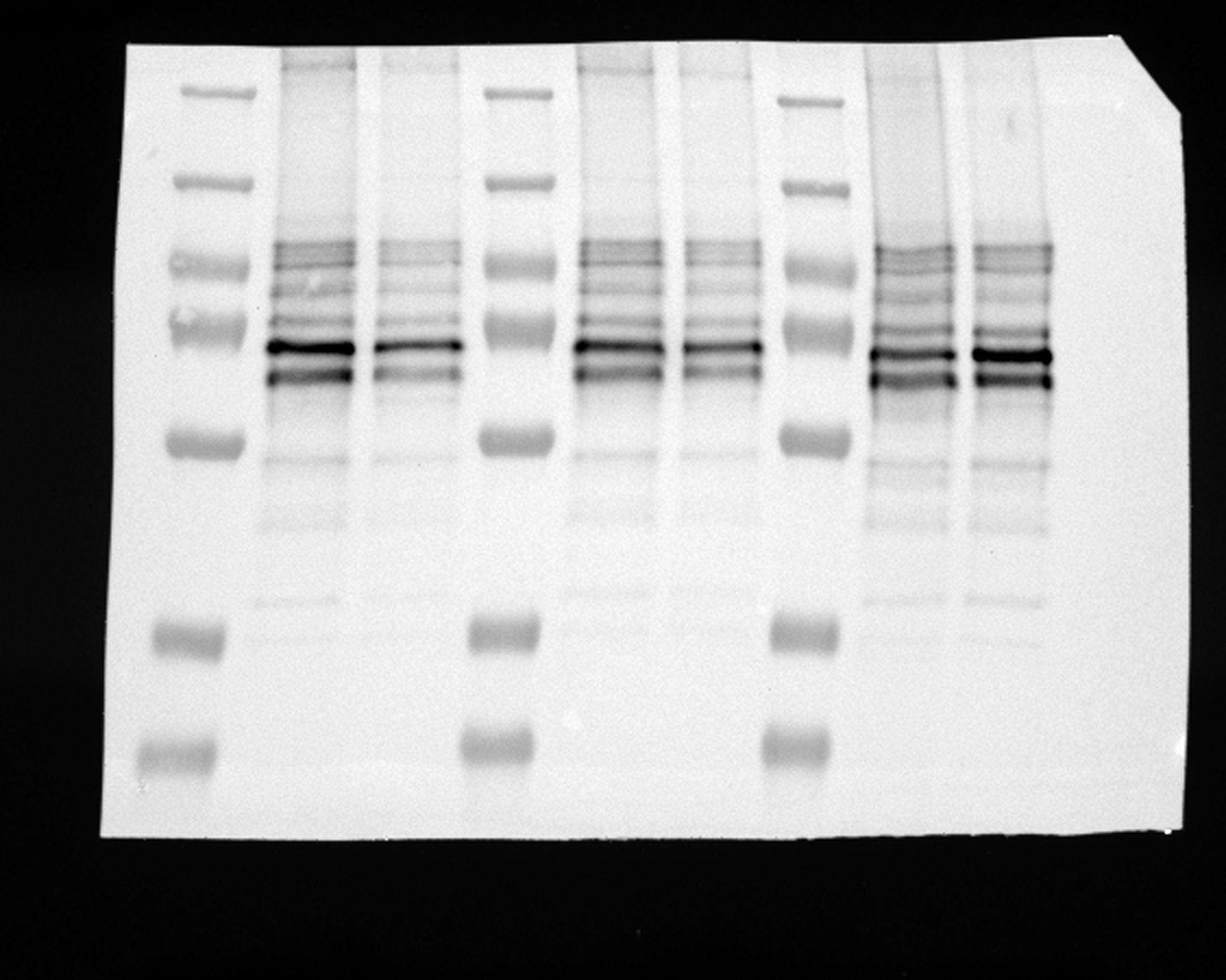

Supplement: Figure 4—figure supplement 1—source data 2. [file elife-108666-fig4-figsupp1-data2.zip › Figure S4 - Source Data 2. Original files for western blot analysis displayed in Figures S4A and S4J/CHEMI_05152025_134549.tif]

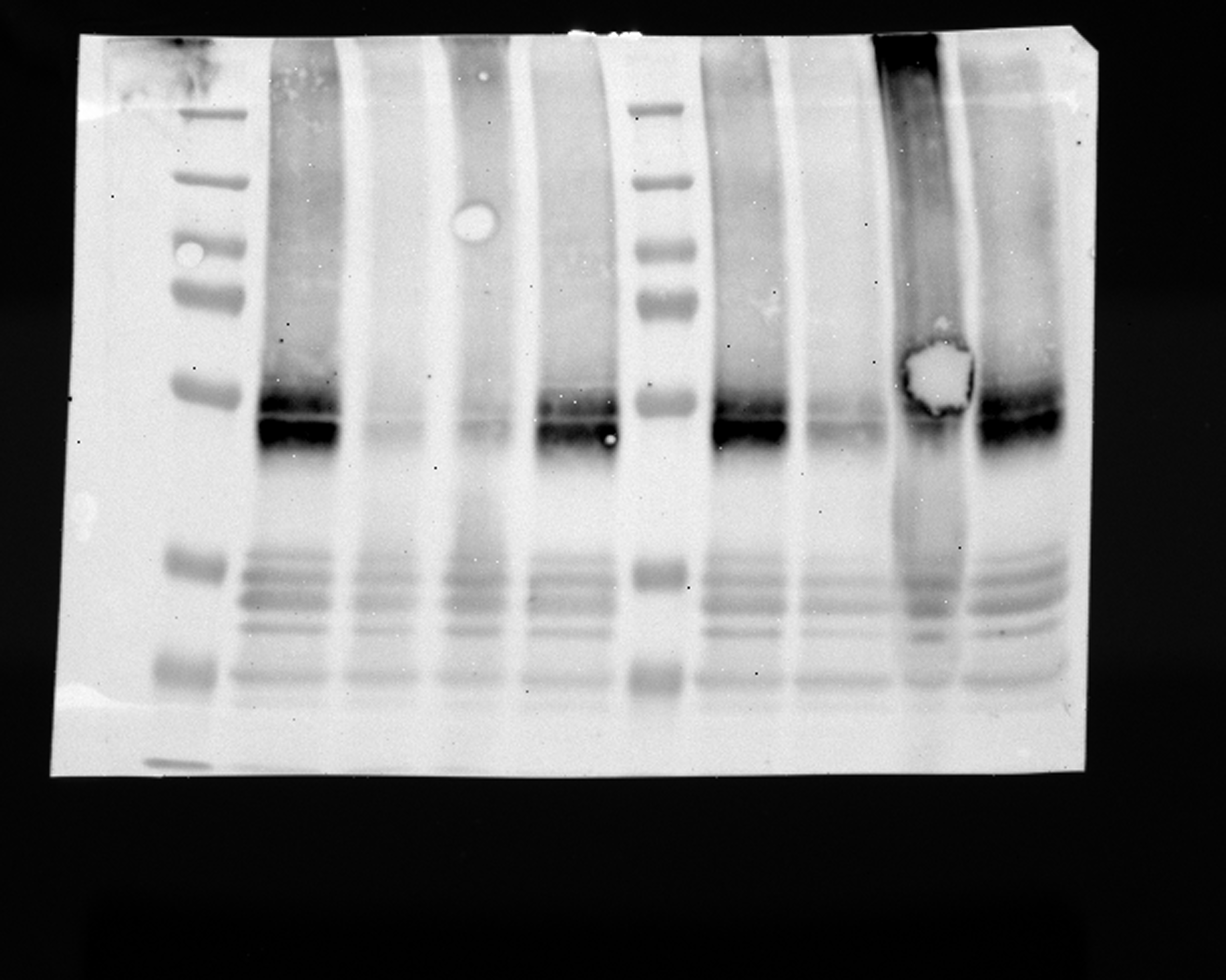

Supplement: Figure 6—source data 2. [file elife-108666-fig6-data2.zip › Figure 6 - Source Data 2. Original files for western blot analysis displayed in Figures 6F and 6H/CHEMI_10122025_145243.tif]

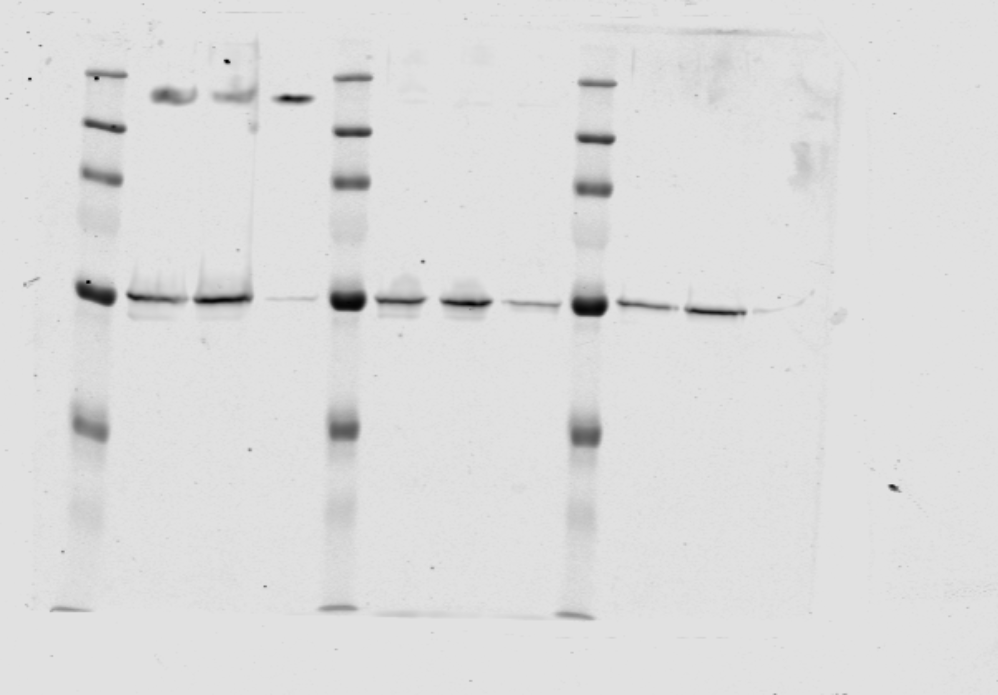

Supplement: Figure 6—source data 2. [file elife-108666-fig6-data2.zip › Figure 6 - Source Data 2. Original files for western blot analysis displayed in Figures 6F and 6H/ZFT 3HA 3_UTR UN_ 50uM ZnSO4_ 5uM TPEN 24h 3 Replicates CDPK1 Loading Control Staining.tif]

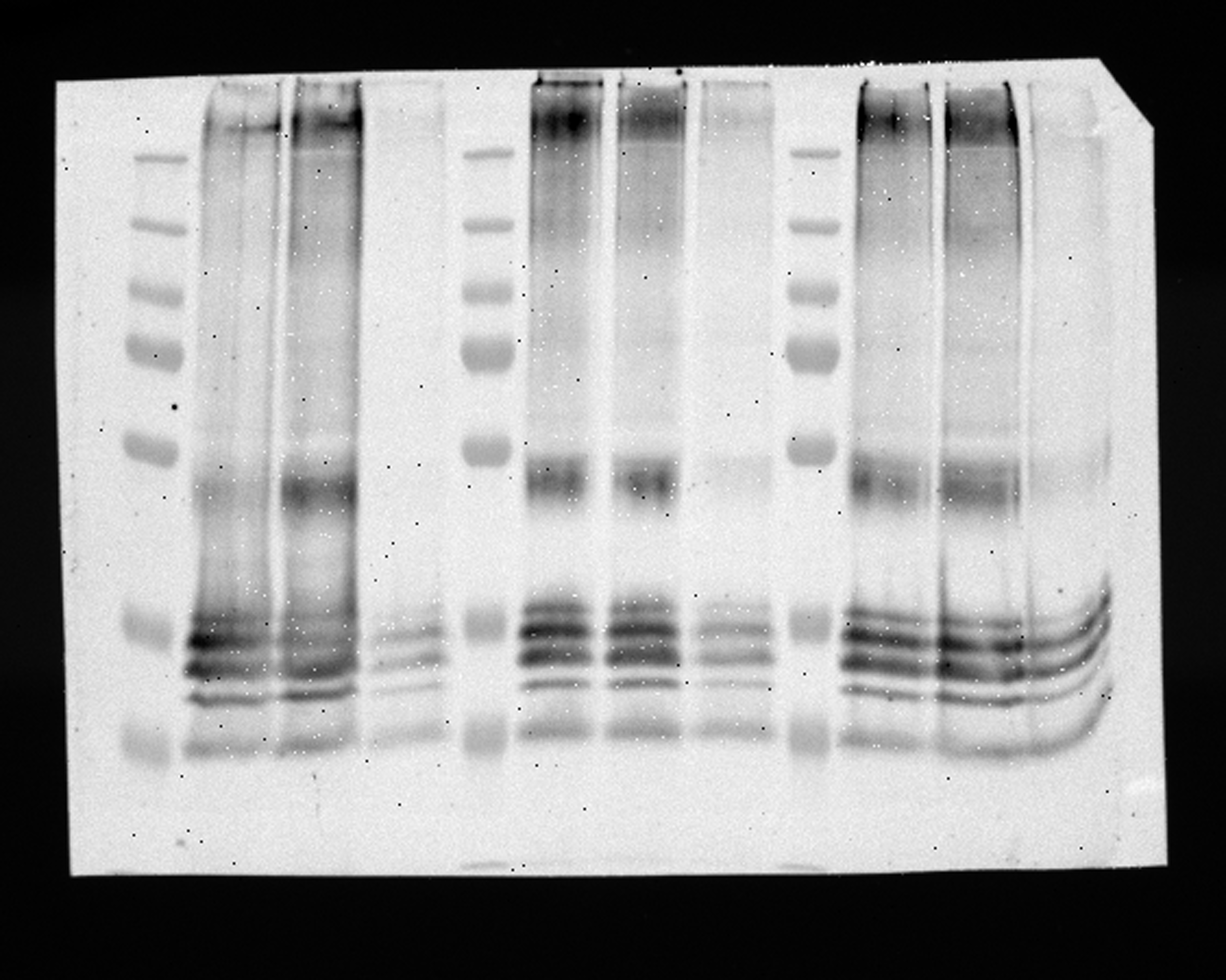

Supplement: Figure 6—source data 2. [file elife-108666-fig6-data2.zip › Figure 6 - Source Data 2. Original files for western blot analysis displayed in Figures 6F and 6H/CHEMI_01082025_142257.tif]
